# The behavioural consequences of dystrophinopathy

**DOI:** 10.1242/dmm.052047

**Published:** 2025-03-03

**Authors:** Minou A. T. Verhaeg, Elizabeth M. van der Pijl, Davy van de Vijver, Christa L. Tanganyika-de Winter, Tiberiu L. Stan, Angel van Uffelen, Luciano Censoni, Maaike van Putten

**Affiliations:** ^1^ Department of Human Genetics, Leiden University Medical Center, 2333 ZA Leiden, The Netherlands; ^2^ The Group for Integrative Neurophysiology, Department of Medical and Translational Biology, Umeå University, 901 87 Umeå, Sweden

**Keywords:** Dystrophin, Cognition, Anxiety, Learning, Spontaneous behavior, Social interaction, *Mdx^5cv^*, *Mdx52* and *DMD-null*

## Abstract

Duchenne muscular dystrophy is a severe neuromuscular disorder, caused by mutations in the *DMD* gene. Normally, the *DMD* gene gives rise to many dystrophin isoforms, of which multiple are expressed in the brain. The location of the mutation determines the number of dystrophin isoforms affected, and the absence thereof leads to behavioral and cognitive impairments. Even though behavioral studies have thoroughly investigated the effects of the loss of Dp427, and to a lesser extent of Dp140, in mice, direct comparisons between models lacking multiple dystrophin isoforms are sparse. Furthermore, a behavioral characterization of the *DMD-null* mouse, which lacks all dystrophin isoforms, has never been undertaken. Using a wide variety of behavioral tests, we directly compared impairments between *mdx^5cv^*, *mdx52* and *DMD-null* mice. We confirmed the role of Dp427 in emotional reactivity. We did not find any added effects of loss of Dp140 on fear, but showed the involvement of Dp140 in spontaneous behavior, specifically in habituation and activity changes due to light/dark switches. Lastly, our results indicate that Dp71/Dp40 play an important role in many behavioral domains, including anxiety and spontaneous behavior.

## Teaser

**Summary:** In mice, the number of dystrophin isoforms lacking in the brain correlates with the severity of behavioral impairments, where those lacking all dystrophin isoforms are most severely affected.

## INTRODUCTION

Duchenne muscular dystrophy (DMD) is a progressive X-linked neuromuscular disorder affecting ∼1 in 5000 newborn males (Mendell et al., 2012). The disorder is characterized by severe muscle wasting, causing loss of muscle function and, eventually, cardiorespiratory failure, resulting in death at ∼30-40 years of age in the Western world (Walter and Reilich, 2017). DMD is caused by mutations in the *DMD* gene, which encodes the protein dystrophin. The gene contains multiple promotors, giving rise to several unique dystrophin isoforms, which differentiate in their expression patterns and functionalities. In muscle, only Dp427m is expressed; however, in the brain, many dystrophin isoforms are expressed in different regions (Doorenweerd et al., 2017).

Many studies have investigated dystrophin expression in the mouse brain (reviewed in Tetorou et al., 2024). Dp427 is mostly expressed in GABAergic inhibitory synapses. More specifically, Dp427c is found in the neurons of the cortex, cornu ammonis regions of the hippocampus, and the cerebellum, whereas Dp427p is selectively expressed in the cerebellum (García-Cruz et al., 2023). Dp140 expression peaks during fetal development; levels in adult animals are relatively low, but present along the walls of blood vessels, specifically the perivascular astrocyte end feet (García-Cruz et al., 2023). Dp71 is the main isoform expressed in astrocytes (García-Cruz et al., 2023; Belmaati Cherkaoui et al., 2021), although recent literature has specified different splice variants playing roles during embryonic and postnatal development (González-Reyes et al., 2024). Dp40 is found in the axon terminals of excitatory cells (García-Cruz et al., 2023).

The location of the mutation in the *DMD* gene determines the number of missing dystrophin isoforms in patients with DMD; mutations at the 5′ end of the gene likely only affect Dp427 expression, whereas mutations closer to the 3′ end are expected to also affect shorter isoforms. Lack of dystrophin in the brain leads to cognitive and behavioral problems in a subset of patients. The severity of these impairments seems to correlate with the number of missing dystrophin isoforms (Ricotti et al., 2016; Taylor et al., 2010; Chamova et al., 2013). Overall, patients with DMD have an IQ that is one standard deviation below the population average (Banihani et al., 2015; Hinton et al., 2007), and a subset of patients suffer from attention-deficit hyperactivity disorder, autism spectrum disorder, obsessive-compulsive disorder, depression, anxiety, inattention, reading deficits and/or epilepsy (Ricotti et al., 2016; Waite et al., 2012; Banihani et al., 2015; Hendriksen and Vles, 2008; Hinton et al., 2006). There is, however, still a lot unknown about how the lack of dystrophin affects the brain.

DMD mouse models have been used to investigate the consequences of dystrophinopathy on the brain. The *mdx* mouse, which lacks Dp427 owing to a nonsense mutation in exon 23, has been most extensively studied (Sicinski et al., 1989). *Mdx* mice have deficits in anxiety (Remmelink et al., 2016a; Saoudi et al., 2021; Vaillend and Chaussenot, 2017; Manning et al., 2014), fear response (Saoudi et al., 2021; Razzoli et al., 2020; Vaillend and Chaussenot, 2017; Sekiguchi et al., 2009; Yamamoto et al., 2010; Lindsay and Russell, 2023; Lindsay et al., 2021), social interaction (Miranda et al., 2015) and, possibly, long-term memory (Bagdatlioglu et al., 2020; Comim et al., 2019; Sesay et al., 1996; Vaillend et al., 2004, 1995; Remmelink et al., 2016a). Direct comparisons of the *mdx* mouse with strains lacking multiple dystrophin isoforms, to investigate correlations between the number of dystrophin isoforms missing and disease severity, are hindered by differences in their genetic backgrounds (C57BL/10ScSnJ for *mdx* versus C57BL/6J for other models). The *mdx^5cv^* mouse, which lacks Dp427 owing to a point mutation in exon 10 (Im et al., 1996), could serve as a viable alternative as it is on a C57BL/6J genetic background. *Mdx^5cv^* mice show abnormalities in social preferences, similar to *mdx* mice (Alexander et al., 2016), but, otherwise, the deficits in *mdx^5cv^* mice remain unclear.

*Mdx52* mice, which lack Dp427, Dp260 and Dp140 owing to deletion of exon 52 (Araki et al., 1997), have been used to study the effects of loss of an additional isoform in the brain. This model shows deficits in anxiety (Saoudi et al., 2021; Hashimoto et al., 2022), social behavior (Hashimoto et al., 2022) and fear learning (Saoudi et al., 2021). Owing to the lack of Dp260, which is normally expressed in the retina, *mdx52* mice also display altered visual processing (Barboni et al., 2021, 2023).

The functions and consequences of the lack of Dp71 and Dp40 have been studied in Dp71-null mice, which show deficits in social interactions, spatial learning, navigation, cognitive flexibility and retinal function (Chaussenot et al., 2019; Helleringer et al., 2018; Miranda et al., 2024; Dalloz et al., 2003). However, because these mice do express the longer dystrophin isoforms, their translational value is limited. The *DMD-null* mouse, which lacks all dystrophin isoforms via a Cre-loxP recombination technique, would be a better model to tackle this issue. They are characterized by restlessness and abnormal maternal behavior (Kudoh et al., 2005), but further knowledge on their behavioral impairments is lacking. Although it is unknown whether the retina of *DMD-null* mice is affected, the lack of Dp260 and Dp71, which are both normally expressed in this tissue, is expected to influence visual processing, at least to a similar extent as in the *mdx52* and Dp71-null mice (Barboni et al., 2021; Dalloz et al., 2003; Howard et al., 1998).

Our study aimed to unravel the consequences of the lack of one, multiple or all brain dystrophin isoforms on behavior in the *mdx^5cv^*, *mdx52* and *DMD-null* models (Table 1). We used an elaborate test setup to study anxiety, fear, social interaction, spontaneous behavior, learning, memory and learning flexibility. This study showed similar alterations in *mdx^5cv^* mice to those previously reported in *mdx* mice (Remmelink et al., 2016a; Saoudi et al., 2021; Vaillend and Chaussenot, 2017; Manning et al., 2014; Razzoli et al., 2020; Sekiguchi et al., 2009; Yamamoto et al., 2010; Lindsay and Russell, 2023; Lindsay et al., 2021), including increased anxiety and fear. Furthermore, we found no additional deficits in *mdx52* mice compared to *mdx^5cv^* mice. Subtle abnormalities in spontaneous behavior were found in *mdx52* mice. Lastly, our study provides the first extensive overview of the behavioral abnormalities in *DMD-null* mice, reporting increased anxiety, fear and deviations in spontaneous behavior in these mice compared to in the other DMD models.

**Table 1.** Overview of mouse models used in this study and their corresponding dystrophin isoforms

Mouse model	Mutation	Dp427	Dp260	Dp140	Dp116	Dp71/Dp40
*Mdx^5cv^*	Point mutation exon 10	−	+	+	+	+
*Mdx52*	Deletion of exon 52	−	−	−	+	+
*DMD-null*	Whole *Dmd* gene removed	−	−	−	−	−

## RESULTS

### Increased anxiety is shown by all DMD models, being most profound in *DMD-null* mice

**Fig. 1.**
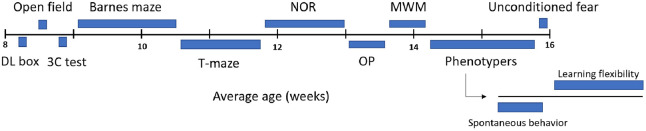
**Study overview.** Mice were included at 8 weeks of age and underwent a variety of behavioral tests for 8 weeks. DL, dark-light box; 3C, three-chamber social interaction; NOR, novel object recognition; OP, object placement; MWM, Morris water maze.

**Fig. 2.**
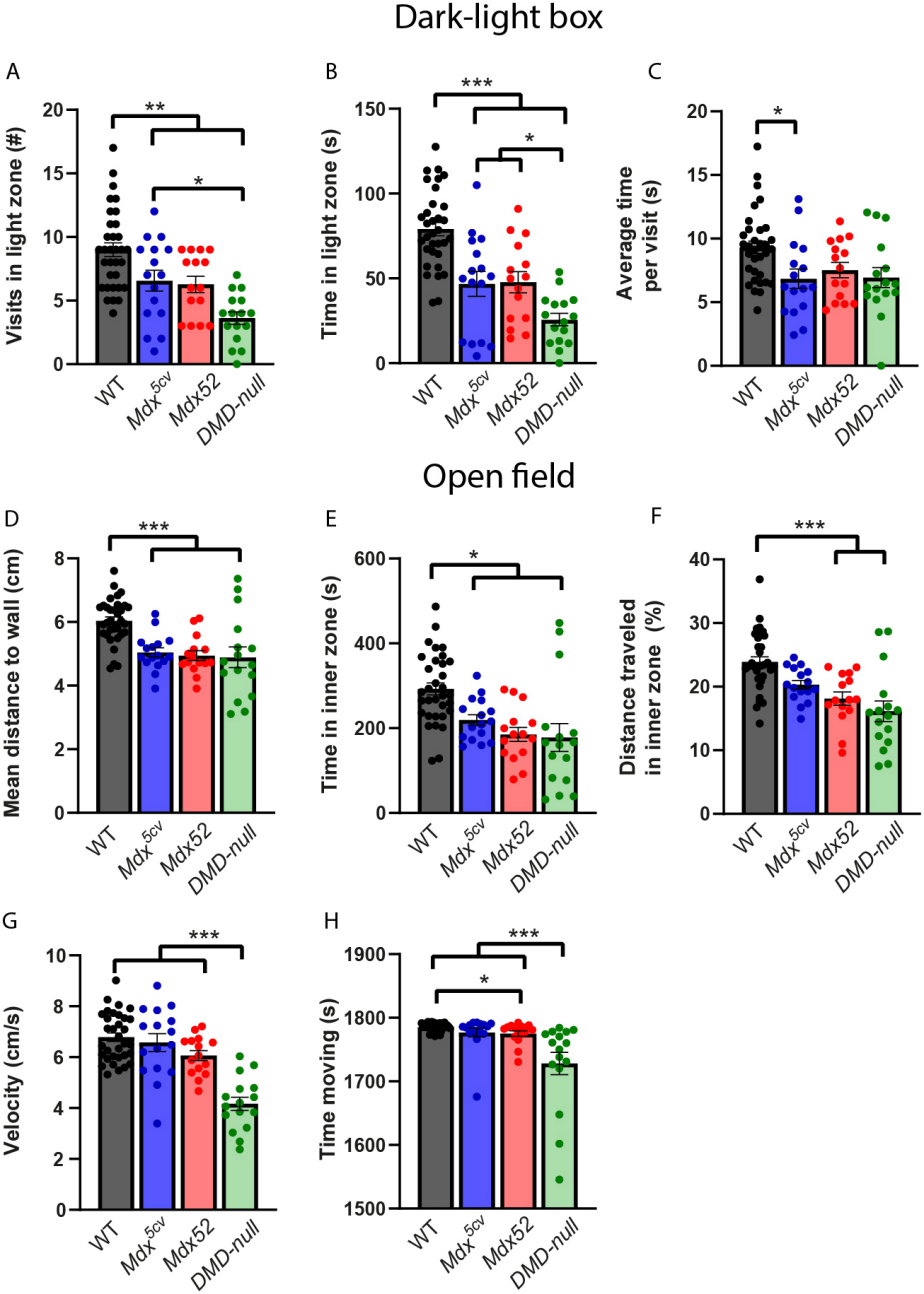
**Anxiety in the dark-light box and open field.** *Mdx^5cv^* (*n*=16), *mdx52* (*n*=15), *DMD-null* (*n*=16) and wild-type (WT) (*n*=33). (A) In the dark-light box, *mdx^5cv^*, *mdx52* and *DMD-null* mice all visited the light compartment less often than WTs (*P=*0.009, *P<*0.001 and *P<*0.001, respectively). *DMD-null* mice visited the light compartment less often than *mdx^5cv^* mice (*P=*0.021). (B) *Mdx^5cv^*, *mdx52* and *DMD-null* mice all spent less time in the light compartment than WTs (all *P<*0.001). *DMD-null* mice spent less time in the light compartment than *mdx^5cv^* and *mdx52* mice (*P*=0.043 and *P*=0.008, respectively). (C) Visits to the light department were on average shorter for *mdx^5cv^* mice than for WTs (*P=*0.022). (D) In the open field test, all DMD models stayed closer to the walls than WTs (all *P<*0.001). (E) *Mdx^5cv^*, *mdx52* and *DMD-null* mice spent less time in the inner zone of the box than WTs (*P=*0.035, *P<*0.001 and *P*<0.001, respectively). (F) *Mdx52* and *DMD-null* mice walked less distance in the inner zone of the box than WT mice (both *P<*0.001). (G) *DMD-null* mice walked slower than WT, *mdx^5cv^* and *mdx52* mice (all *P<*0.001). (H) *DMD-null* mice spent less time moving than WT, *mdx^5cv^* and *mdx52* mice (all *P<*0.001). *Mdx52* mice moved less than WTs (*P=*0.017). *P<0.05, **P<0.01, ***P<0.001 (we refer the reader to Table S2 for an overview of the statistical tests performed).

Behavior of the DMD mice was assessed using a variety of behavioral tests (Fig. 1). To assess anxiety, the dark-light box and the open field tests were used. In the dark-light box, mice were allowed to move freely between the light and dark compartments for 5 min (Fig. 2A-C). *Mdx^5cv^*, *mdx52* and *DMD-null* mice all visited the light compartment less often than wild-type (WT) mice (*P*=0.009, *P<*0.001 and *P*<0.001, respectively) and spent less time in the light compartment (all *P*<0.001) (Fig. 2A,B). Additionally, *DMD-null* mice visited the light compartment less often than *mdx^5cv^* mice (*P*=0.021) and spent less time in the light compartment than *mdx^5cv^* and *mdx52* mice (*P=*0.043 and *P*=0.008, respectively). Only *mdx^5cv^* mice spent less time in the light compartment per visit than WTs (*P=*0.022) (Fig. 2C).

The open field box was used to further assess anxiety (Fig. 2D-H). Both location and locomotion were tracked during this 30 min test. Overall, *mdx^5cv^*, *mdx52* and *DMD-null* mice stayed closer to the walls of the box (all *P<*0.001), and spent less time in the inner zone of the box (*P=*0.035, *P<*0.001 and *P*<0.001, respectively), than WT mice (Fig. 2D,E). *Mdx52* and *DMD-null* mice walked relatively less distance in the inner zone of the box than WTs (both *P*<0.001) (Fig. 2F). Additionally, *DMD-null* mice walked slower (all *P<*0.001) and spent less time moving (all *P<*0.001) than WT, *mdx^5cv^* and *mdx52* mice (Fig. 2G,H). *Mdx52* mice also spent less time moving than WTs (*P=*0.017). Additionally, locomotor behavior was analyzed for changes over time (Fig. S3). Apart from a lack of initial activity found in all DMD models in the first 5 min time bin, no differences could be found between the groups over time. Overall, all DMD models showed increased anxiety; however, the anxious behavior seems most aggravated in *DMD-null* mice.

### The strong fear response in *mdx^5cv^* and *mdx52* mice is further aggravated in *DMD-null* mice

**Fig. 3.**
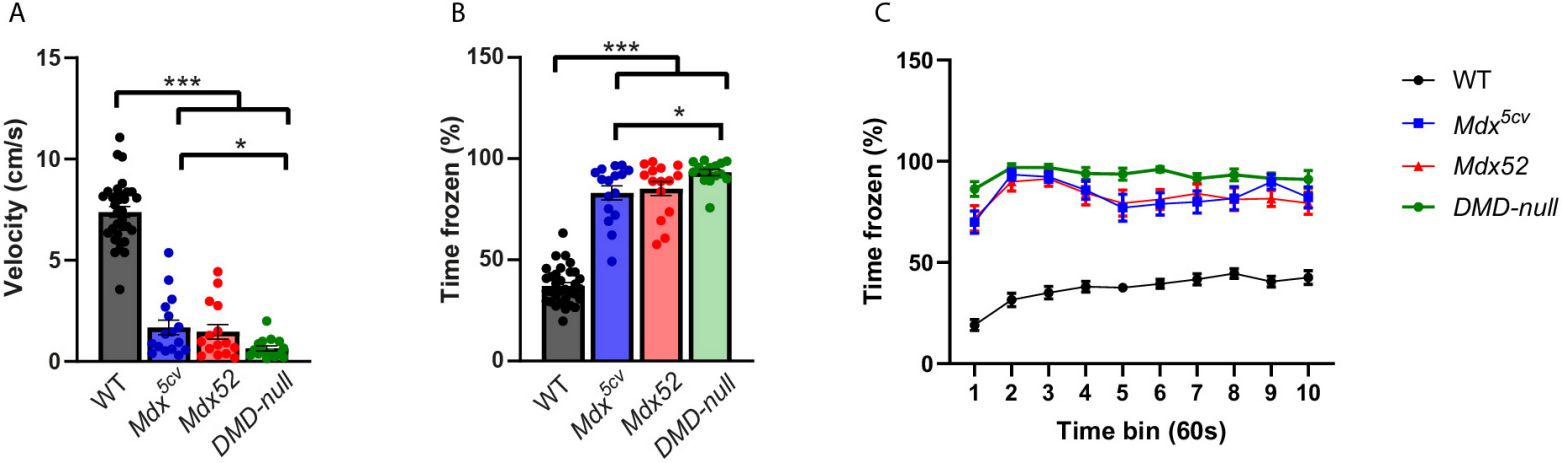
**Unconditioned fear response after a short restraint.** *Mdx^5cv^* (*n*=16), *mdx52* (*n*=15), *DMD-null* (*n*=16) and WT (*n*=33). (A) In all DMD models, walking velocity was decreased compared to that in WTs (all *P<*0.001). *DMD-null* mice showed a further decrease compared to *mdx^5cv^* mice (*P*=0.012). (B) Freezing time was increased in all DMD models (all *P*<0.001). *DMD-null* mice spent more time frozen than *mdx^5cv^* mice (*P*=0.019). (C) Freezing time per time bin of 60 s. All DMD strains show a different pattern over time compared to WTs (*P<*0.001), but no differences were found between the DMD strains*.* *P<0.05, ***P<0.001 (Mann–Whitney test).

To assess unconditioned fear, mice were restrained and held upside down for 15 s before being put in the open field box. In all DMD models, walking velocity was severely decreased (all *P<*0.001), and freezing time was markedly increased (all *P*<0.001), compared to that in WTs (Fig. 3A,B). Additionally, *DMD-null* mice walked slower (*P*=0.012) and spent more time frozen (*P=*0.019) than *mdx^5cv^* mice. A trend was also observed in the same direction when time spent frozen was compared to that in *mdx52* mice (*P=*0.050). To determine whether freezing behavior was time dependent in the strains, the percentage of time frozen was calculated in time bins of 60 s. All DMD models behaved differently over time compared to WTs (*P<*0.001), but no differences were found between DMD strains over time (Fig. 3C). *Mdx^5cv^*, *mdx52* and *DMD-null* mice all exhibited a very strong increase in fear response, with *DMD-null* mice showing more excessive freezing than the other models.

### Results on social interactions remain inconclusive owing to lack of preference in WT mice

Social interaction was analyzed using the three-chamber social interaction paradigm, in which experimental mice could interact with other mice that were contained in a tube. After habituation, the animals could choose to investigate either an object or a novel mouse (Fig. S4A). Directly after this trial, the mice were given a new choice – this time between interaction with a novel mouse or a familiar mouse, which was used in the previous trial (Fig. S4B). Unfortunately, in both trials, the WT mice did not show any preference for one of the targets, meaning that they spent an equal amount of time in both tubes in each trial (Fig. S4C). Furthermore, no differences were found between the groups. Notably, *mdx52* mice spent more time interacting with the mouse in the first trial, showing a preference for the investigation of the mouse versus the object, which was significantly different from chance level (*P=*0.024). *DMD-null* mice interacted more with the novel mouse than with the familiar one (*P=*0.008). However, owing to the lack of preferences observed in WTs, data on DMD mice are inconclusive in terms of deficits in socialization or social novelty seeking.

### Spatial memory is dependent on stress in *mdx52* and *DMD-null* mice, with small deviations in reversal learning in *DMD-null* mice

**Fig. 4.**
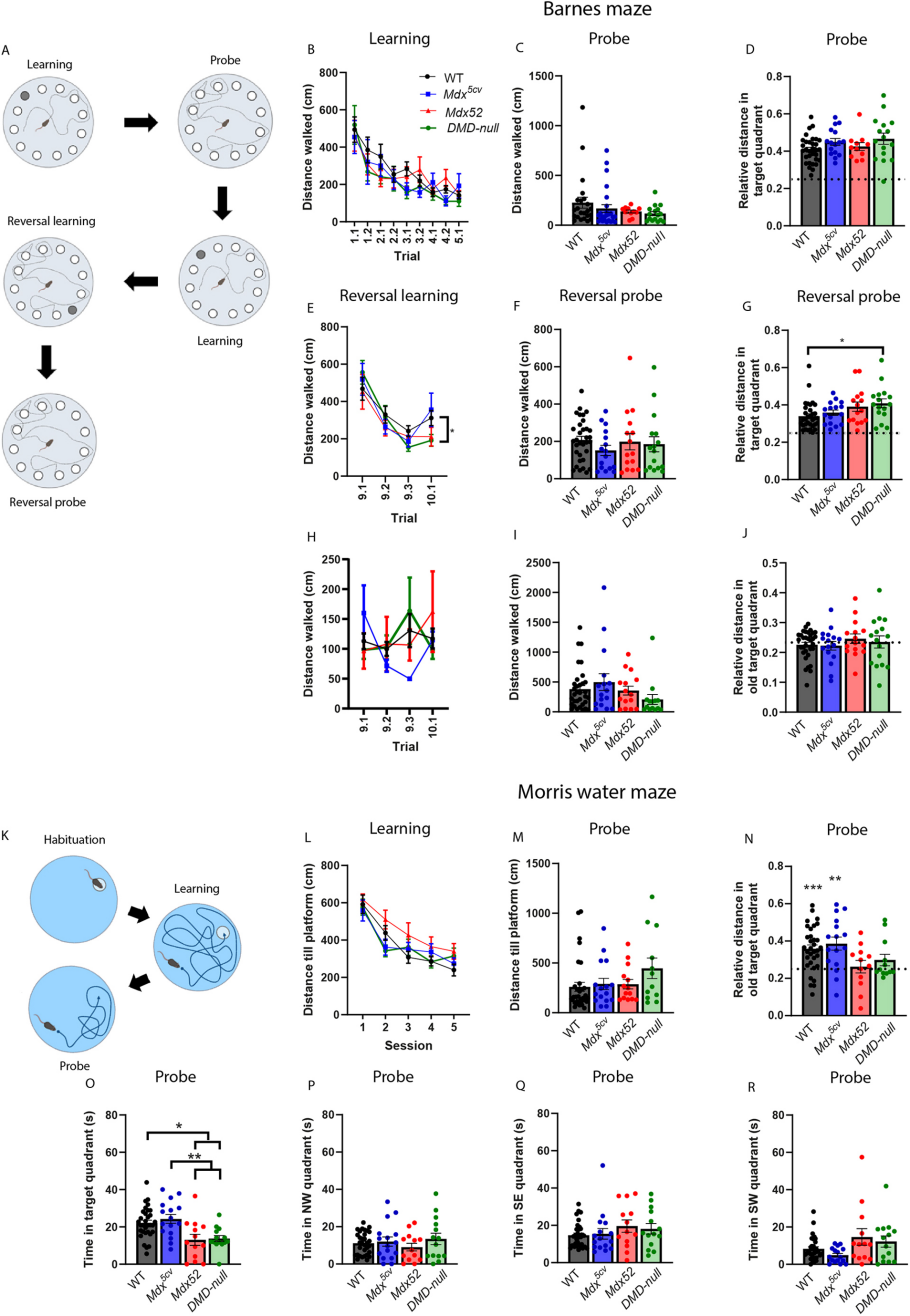
**Spatial learning and memory in the Barnes maze and Morris water maze.** (A) Schematic of the Barnes maze protocol. *Mdx^5cv^* (*n*=16), *mdx52* (*n*=15), *DMD-null* (*n*=16) and WT (*n*=33). Created in BioRender by Verhaeg, M. (2025). https://BioRender.com/p79/689. This figure was sublicensed under CC-BY 4.0 terms. (B) Distance traveled to reach the platform during the acquisition learning. No differences were found between groups. (C) All groups traveled approximately equal distances to reach the platform zone during the probe trial. (D) No significant differences were found between groups in the relative distance traveled in the target quadrant. All groups traveled more distance in this quadrant compared to chance level (0.25, indicated by the dotted line) (all *P*<0.001). (E) *DMD-null* mice showed increased distance traveled to the new platform location during reversal learning compared to WTs (*P*=0.015). (F) No differences were found between groups in the distance traveled to reach the new platform zone during the reversal probe trial. (G) *DMD-null* mice showed a higher relative distance traveled in the new target quadrant compared to WTs (*P*=0.033). All groups performed above chance level (all *P*<0.001, indicated by the dotted line). (H,I) No differences were found between groups in distance traveled until reaching the old platform location during the reversal learning (H) or the reversal probe (I). (J) No differences were found between groups in the relative distance traveled in the old target quadrant. None of the groups differed from chance level. (K) Schematic of the Morris water maze protocol. *Mdx^5cv^* (*n*=16), *mdx52* (*n*=13), *DMD-null* (*n*=11) and WT (*n*=33). Created in BioRender by Verhaeg, M. (2025). https://BioRender.com/p79/689. This figure was sublicensed under CC-BY 4.0 terms. (L) No differences were found between groups in the distance they needed to swim to find the platform. (M) No differences were found in the distance swam until reaching the platform during the probe trial. (N) No differences were found between groups in the relative distance swam in the target quadrant. Only the performances of WT and *mdx^5cv^* mice differed from chance level, indicated by the dotted line at 0.25 (*P*<0.001 and *P*=0.002, respectively). (O) *Mdx52* and *DMD-null* mice spent less time in the target quadrant than *mdx^5cv^* (*P=*0.005 and *P=*0.009, respectively) and WT (*P=*0.012 and *P=*0.022, respectively) mice. (P-R) No difference was found in the time spent in any of the other quadrants. *P<0.05, **P<0.01, ***P<0.001 (we refer the reader to Table S2 for an overview of the statistical tests performed).

Spatial navigation, memory and flexibility were assessed using the Barnes maze and the Morris water maze. Owing to decreased walking and swimming velocities in the DMD mouse models (Fig. S5A,B), latency to reach the target was excluded as a parameter; instead, distance traveled until reaching the target was used. The Barnes maze consists of a large wooden circular platform with 12 holes. An escape box could be placed under any of the holes, and the mice needed to navigate to the correct hole using external cues. The protocol consisted of 5 learning days, a probe trial, 2 rest days, another learning day, 2 days of reversal learning, and a reversal probe trial (Fig. 4A). No differences were found in the distance the mice walked to find the target location during either the learning or probe trials (Fig. 4B,C), or in the relative distance walked in the target quadrant during the probe trial (Fig. 4D). All groups walked significantly greater distance in the target quadrant compared to chance level (all *P<*0.001). No deviations were found in the time spent at the target hole during the probe test (Fig. S5C).

Surprisingly, when reversal learning was started, *DMD-null* mice walked a shorter distance to reach the new target hole (*P=*0.015) compared to WTs (Fig. 4E). No differences were found in distance traveled to the target hole during the reversal probe trial even though all groups walked relatively greater distance in the target quadrant compared to chance level (all *P<*0.001). *DMD-null* mice also walked relatively greater distance in the new target quadrant compared to WTs (*P=*0.033) (Fig. 4F,G). No differences were found between groups in terms of distance traveled to the old target location in the learning or probe trials (Fig. 4H,I). Animals did not differ in relative walking distance traveled in the old target quadrant (Fig. 4J), or in the interaction time with any of the holes (Fig. S5D), indicating no change in the interference of the earlier training on the behavior of the animals.

During the Morris water maze, spatial learning and memory were assessed in a more stressful environment due to the high demand of the test on muscle function (Fig. 4K). *DMD-null* mice, in particular, struggled with the protocol, and, eventually, two *mdx52* and five *DMD-null* mice had to prematurely stop the acquisition learning owing to inability to keep their head above the water. No differences were found in the distance the mice traveled until reaching the platform during the learning (Fig. 4L) and probe (Fig. 4M) trials. No differences were found between groups when comparing the relative distance traveled in the quadrant in which the platform was located (Fig. 4N). However, only WT and *mdx^5cv^* mice showed a distance traveled significantly different from the chance level of 0.25 (*P<*0.001 and *P=*0.002, respectively). Furthermore, *mdx52* and *DMD-null* mice spent less time in the target quadrant than *mdx^5cv^* (*P=*0.005 and *P=*0.009, respectively) and WT (*P=*0.012 and *P=*0.022, respectively) mice, but no differences were found in time spent in any of the other quadrants (Fig. 4O-R). Overall, no deficits in spatial navigation could be found. *DMD-null* mice did, however, seem to perform better than WTs during the reversal learning tests. Results on memory retention are conflicting between the Morris water maze and the Barnes maze, possibly owing to the difference in demand in motor function.

### Spatial and non-spatial recognition memory are not affected in any of the DMD models

To assess recognition memory for both short- and long-term delay, mice underwent the novel object recognition, object placement and T-maze tests. During the object recognition task, mice were exposed to three objects. After a delay of 10 min or 24 h, one of the objects was replaced by a novel object. The discrimination index (DI) was calculated for the amount of time the mice spent exploring the new or old objects. All mice showed a preference for the new object during both delays, indicated by a DI above chance level, but no differences were found in DI between any of the groups for either time delay (Fig. S6A).

During the object placement task, two identical objects were placed in the box, together with spatial visual cues. After a delay of 10 min or 24 h, one of the objects was moved to a different corner, and the DI was calculated for the new versus old placement. No differences were found in DI scores; however, even though all mice showed a preference for the displaced object after a 10 min delay, only WT mice showed a significant difference compared to chance level after a 24 h delay (*P=*0.014) (Fig. S6B).

Mice were subjected to the T-maze test two times, once with a 6 h delay and once with a 24 h delay. When reintroduced into the maze after the delay, the portion of time spent in the target arm versus the total time in either arm was calculated (DI). WT mice did not show any preference for the novel arm in either delay (Fig. S6C). Furthermore, no differences were found in the DI for any of the groups at either the 6 h or 24 h delay, and no differences were found in the percentage of mice that chose the target arm as a first entry (alternation) in any of the groups at either delay (Fig. S6C). Notably, *mdx52* and *DMD-null* mice performed above chance level in terms of percentage of alternation (*P*=0.02 and *P*=0.046, respectively). Because WT mice did not show significant alteration, the results of this test are inconclusive. However, based on the other tests, none of the dystrophin isoforms seem to have a substantial effect on short- or long-term recognition memory.

### Initial reversal learning deficits found in *mdx^5cv^* and *mdx52* mice seem to be absent in *DMD-null* mice

**Fig. 5.**
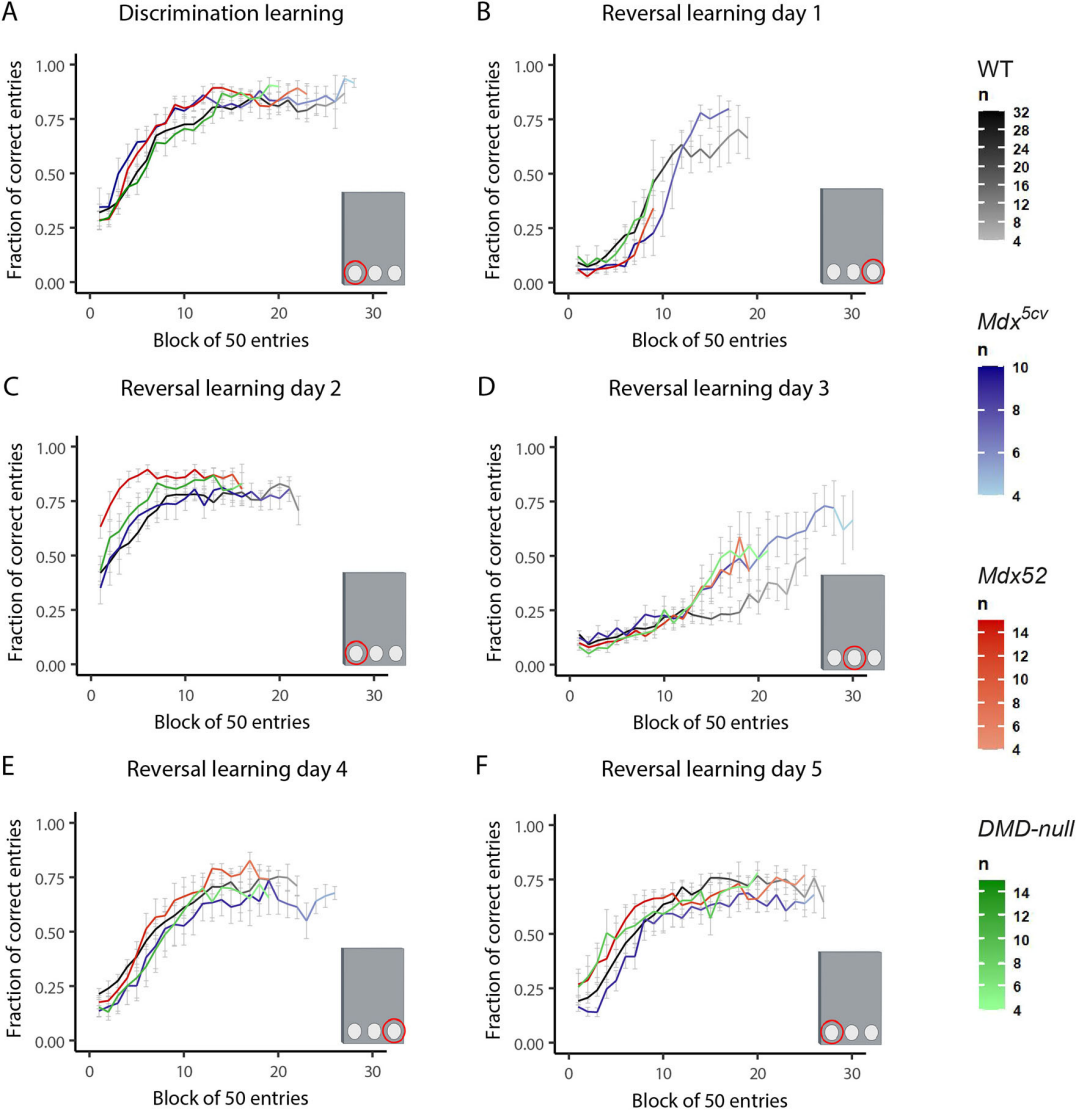
**Serial reversal learning with food reward in PhenoTyper cages.** *Mdx^5cv^* (*n*=9-10), *mdx52* (*n*=14), *DMD-null* (*n*=10-15) and WT (*n*=32-33). (A) Discrimination learning (left target). Both *mdx^5cv^* and *mdx52* mice showed steeper learning curves than those of WTs (*P<*0.001 and *P=*0.011, respectively) and *DMD-null* mice (*P<*0.001 and *P=*0.018, respectively). (B) Reversal day 1 (right target). *Mdx^5cv^* mice showed decreased learning compared to WTs (*P*=0.017). (C) Reversal day 2 (left target). *Mdx52* mice showed increased performance compared to WTs and *mdx^5cv^* mice (*P<*0.001 and *P=*0.013, respectively). (D) Reversal day 3 (middle target). *Mdx52* mice showed increased learning compared to WTs (*P=*0.018). (E) Reversal day 4 (right target). No differences were found between groups. (F) Reversal day 5 (left target). No differences were found between groups. All graphs were cut off at *n=*3 for visual purposes. Cartoons were created in BioRender by Verhaeg, M. (2025). https://BioRender.com/e60i623. This figure was sublicensed under CC-BY 4.0 terms.

To further assess flexibility of memory in an environment with minimal external influences, mice were individually housed in PhenoTyper cages. A cognition wall with three holes was placed in one corner with a pellet dispenser at the back, providing the only food available for the mice. A food pellet was given if the mouse went through the correct entrance five times (which did not have to be consecutive). Weight loss was monitored closely (Fig. S2B), and mice were taken out of the experiment prematurely in the case of more than 20% weight loss since the start of the task or tonic immobility. To adjust for differences in activity between the strains, learning curves were calculated per amount of entries rather than time. Fractions of correct entries, in bins of 50 entries, were calculated for the target hole (Fig. 5) and the perseverative errors (meaning the mouse entered the target from the previous day) and neutral errors (meaning the mice entered the third hole, which was not the current target or the target of the previous day) (Fig. S7). As not all mice performed the same amount of entries, the amount of animals on which the curves are based decreases as the curves progress. For visual purposes, graphs are depicted only as long as the average curve is calculated from *n>*3 mice per group. During the discrimination learning phase in the first 2 days, the left entrance was deemed the target entrance; afterwards, the target entrance changed every 24 h during the reversal learning phase. Both *mdx^5cv^* and *mdx52* mice learned the correct target hole faster than WT (*P*<0.001 and *P=*0.011, respectively) and *DMD-null* (*P<*0.001 and *P*=0.018, respectively) mice (Fig. 5A). After the first change in target entrance, *mdx^5cv^* and *mdx52* mice both showed a decreased learning curve compared to that of WTs; however, this was only significant in *mdx^5cv^* mice (*P=*0.017) (Fig. 5B). Notably, *mdx52* and *DMD-null* mice were less active, only showing activity for nine or ten bins of 50 entries. Activity per hour was plotted to check for any abrupt changes in activity, but no sudden changes in activity could be observed, only a continuously lower activity level of the mice throughout the day (Fig. S8). During the second switch, the target hole moved back to the original location. *Mdx52* mice showed increased performance compared to that of WT and *mdx^5cv^* mice (*P<*0.001 and *P*=0.013, respectively) (Fig. 5C). This increase in performance is most likely due to the decreased activity on the previous day, preventing *mdx52* mice from learning the new location during the first reversal day. Even though *DMD-null* mice had similar levels of activity as the *mdx52* mice, this did not lead to increased performance for these mice. During the third target switch, the target entrance changed to the middle entrance for the first time. Interestingly, *mdx52* mice performed better than the WT mice (*P*=0.018), and the *mdx^5cv^* and *DMD-null* mice also showed a similar trend (*P=*0.05 and *P*=0.066, respectively) (Fig. 5D). During reversal learning day 4 (target hole right) and day 5 (target hole left), no differences were found between the groups (Fig. 5E,F).

### *DMD-null* mice show several deviations in spontaneous behavior, pointing to more restless type of activity; small alterations are also found in *mdx52* mice

**Fig. 6.**
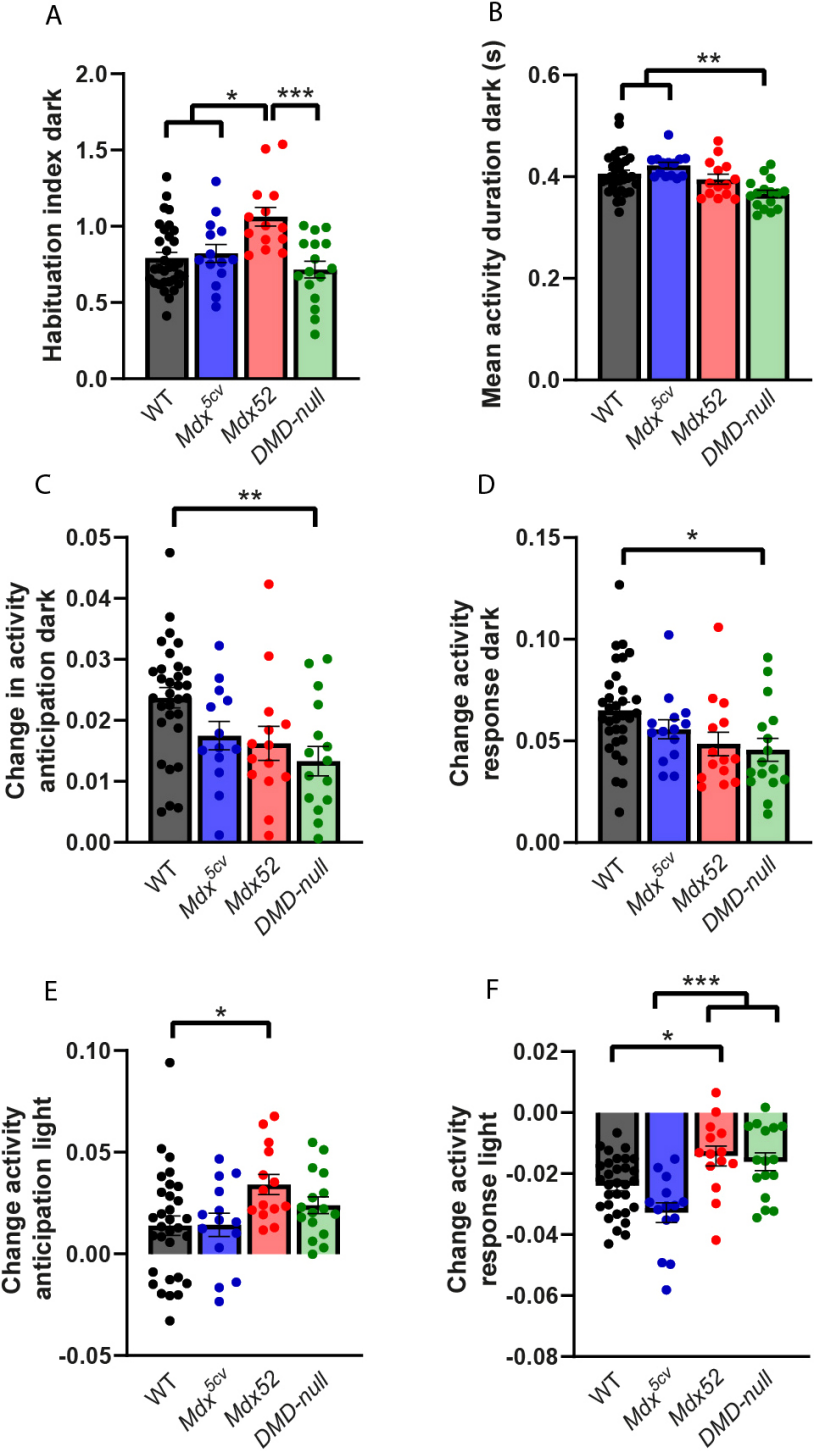
**Spontaneous behavior in the PhenoTyper cages: activity-based behavior.** *Mdx^5cv^* (*n*=14), *mdx52* (*n*=14), *DMD-null* (*n*=16) and WT (*n*=33). (A) *Mdx52* mice showed a higher habituation index during the dark phase compared to WT, *mdx^5cv^* and *DMD-null* mice (*P<*0.001, *P=*0.025 and *P*<0.001, respectively). (B) Mean activity duration during the dark phase was lower in *DMD-null* mice than in WT and *mdx^5cv^* mice (*P=*0.002 and *P<*0.001, respectively). (C) In *DMD-null* mice, activity in anticipation of the dark phase was less changed than in WT mice (*P*=0.004). (D) *DMD-null* mice showed less change in activity in response to the start of the dark phase than WTs (*P=*0.023). (E) *Mdx52* mice showed a higher increase in activity in anticipation of the start of the light phase than WTs (*P=*0.032). (F) *Mdx52* mice showed a lower decrease in activity change in response to the start of the light phase than WT and *mdx^5cv^* mice (*P=*0.037 and *P<*0.001, respectively). *DMD-null* mice also showed less of a decrease in activity than *mdx^5cv^* mice (*P<*0.001). *P<0.05, **P<0.01, ***P<0.001 (one-way ANOVA with post-hoc Tukey).

Spontaneous behavior was assessed during 3 days of continuous monitoring in the PhenoTyper cages, with minimal external influences and no direct handling. Twenty parameters of spontaneous behavior concerning activity, changes in activity due to light switches, sheltering behavior and movement patterns were analyzed as described in Loos et al. (2014) (Fig. 6). When possible, parameters were measured during day 3 to minimize the impact of the new environment.

Four parameters showed significant differences between WT groups (Fig. S9). These parameters included change in activity in anticipation to the dark phase, activity duration during the light phase, dark-light activity index and long arrest threshold. In one of these parameters, change in activity in anticipation to the dark phase, results were similar between groups whether or not the DMD models were compared to the individual or the pooled WT group (Fig. S9A). Therefore, this parameter was not excluded from analysis. The other three parameters only showed significant differences between DMD models and WT mice when the WT group was pooled, but not when compared to their own WTs, and were therefore excluded from analysis to prevent false positives (Fig. S9B-D).

*Mdx52* mice showed an increased habituation index compared to that of WT, *mdx^5cv^* and *DMD-null* mice (*P<*0.001, *P=*0.025 and *P*<0.001, respectively) (Fig. 6A). The *mdx52* mice had an average habituation index close to 1, meaning that they had similar activity levels during the first and third dark phase; mice with a habituation index below 1 had lower activity on the third compared to the first dark phase (Loos et al., 2014). During the third dark phase, the average duration of activity was lower in *DMD-null* mice than in WT and *mdx^5cv^* mice (*P=*0.002 and *P<*0.001, respectively) (Fig. 6B). To assess differences in the anticipation or the response of a switch between dark and light phases, the average activity during the last 2 h of the original phase (when in anticipation) or during the first 2 h of the next phase (when in response) was calculated, and the baseline – the average of the eighth, ninth and tenth hour of activity of the original phase – was subtracted. During anticipation of the dark phase and in response to the start of the dark phase, *DMD-null* mice showed less of an increase in activity than WTs (*P*=0.004 and *P=*0.023, respectively) (Fig. 6C,D). It should be noted that for the change in activity in anticipation of the dark phase, significant differences were found between WT groups; however, when they were split, *DMD-null* mice still showed a significantly smaller change in activity compared to their own WT group (*P*=0.020) (Fig. S9A). Change in activity in anticipation of the light phase was increased in *mdx52* mice compared to that in WTs (*P=*0.032) (Fig. 6E). After the light phase started, *mdx52* mice showed decreased change in activity compared to WT and *mdx^5cv^* mice (*P=*0.037 and *P<*0.001, respectively) (Fig. 6F). In *DMD-null* mice, the decrease in activity was also less pronounced than in *mdx^5cv^* mice (*P<*0.001).

**Fig. 7.**
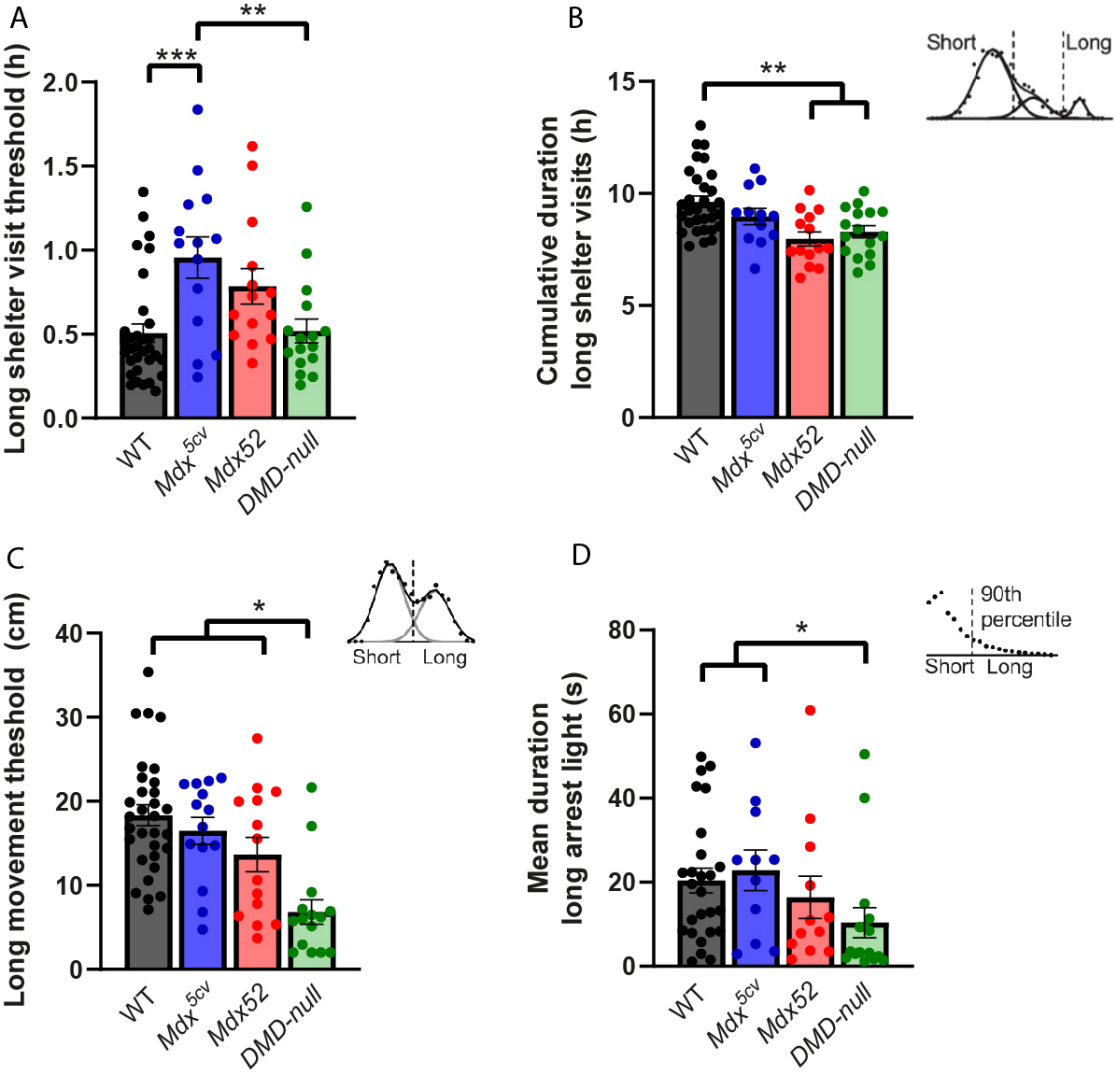
**Spontaneous behavior in the PhenoTyper cages: sheltering behavior, movement and arrest.** *Mdx^5cv^* (*n*=14), *mdx52* (*n*=14), *DMD-null* (*n*=16) and WT (*n*=33). (A) The long shelter visit threshold was increased in *mdx^5cv^* mice compared to that in WT and *DMD-null* mice (*P<*0.001 and *P*=0.007, respectively). (B) Both *mdx52* and *DMD-null* mice showed a lower cumulative duration of shelter visits above the long shelter visit threshold compared to that in WTs (*P<*0.001 and *P=*0.009, respectively). (C) The long movement threshold was lower in *DMD-null* mice than in WT, *mdx^5v^* and *mdx52* mice (*P<*0.001, *P<*0.001 and *P=*0.027, respectively). (D) *DMD-null* mice had a lower duration of arrests above the threshold than WT and *mdx^5cv^* mice (*P=*0.014 and *P=*0.009, respectively). *P<0.05, **P<0.01, ***P<0.001 (we refer the reader to Table S2 for an overview of the statistical tests performed).

To assess more complex patterns of sheltering behavior, the duration of each shelter visit was measured, log2 transformed and plotted in a frequency plot per mouse so that three Gaussian curves could be fitted over the data. These curves were used to determine the short shelter visit threshold (90th percentile of the first curve) and the long shelter visit threshold (intersection between the second and third curve). Significant differences were found in the long shelter visit threshold of *mdx^5cv^* mice compared to that of WT and *DMD-null* mice (*P<*0.001 and *P=*0.007, respectively) (Fig. 7A). However, when calculating the cumulative duration of long shelter visits, *mdx52* and *DMD-null* mice, but not *mdx^5cv^* mice, showed a decrease in long shelter visit duration compared to that of WTs (*P<*0.001 and *P=*0.009, respectively) (Fig. 7B).

To assess movement patterns, the distance of each movement was measured, log2 transformed and plotted in a frequency plot to fit two Gaussian curves over the data. The intersection between the two curves determined the long movement threshold. *DMD-null* mice showed a lower long movement threshold than that of WT, *mdx^5cv^* and *mdx52* mice (*P<*0.001, *P<*0.001 and *P=*0.027, respectively) (Fig. 7C). Lastly, arrest patterns were analyzed by calculating the duration of each arrest. A long arrest threshold was determined by the 90th percentile of data. The threshold did not differ significantly between strains, but, when looking at the arrests that were above the threshold, the average duration of these arrests was lower in *DMD-null* mice than in WT and *mdx^5cv^* mice (*P*=0.014 and *P=*0.009, respectively) (Fig. 7D).

Overall, mostly *DMD-null* mice showed changes in spontaneous activity, but small deviations could be found in all models.

## DISCUSSION

The consequences of the lack of Dp427 in the brain have been thoroughly investigated (Remmelink et al., 2016a; Saoudi et al., 2021; Vaillend and Chaussenot, 2017; Manning et al., 2014; Razzoli et al., 2020; Sekiguchi et al., 2009; Yamamoto et al., 2010; Lindsay and Russell, 2023), and, in recent years, our knowledge on the consequences of lack of Dp140 has expanded (Saoudi et al., 2021; Hashimoto et al., 2022). However, many studies focused on a single DMD mouse model, making it difficult to directly compare the extent of behavioral alterations between strains lacking one or multiple dystrophin isoforms. Furthermore, behavioral characterization of *DMD-null* mice had not been undertaken before, limiting the knowledge on the consequences of the additional loss of Dp71 and Dp40. This study aimed to directly compare different behavioral aspects between three DMD mouse models to gain new insights into the cognitive deficits of these models.

### Anxiety increases due to the lack of Dp427, Dp140, Dp71 and Dp40

One of the most studied behaviors in DMD mouse models is anxiety. Studies on mice lacking Dp427 (*mdx* and *mdx^5cv^*) suggested that these models have a subtle and somewhat borderline deficit in anxiety, while mice lacking Dp427 and Dp140 show a more profound anxiety response (Saoudi et al., 2021). Although differences between our *mdx^5cv^* and *mdx52* mice were subtle, multiple parameters did show a stepwise pattern in which the *mdx52* mice were consistently slightly more anxious than *mdx^5cv^* mice. The ability to detect anxious behavior can be highly dependent on the protocol and surroundings; possibly, variations in materials could affect the chance of finding subtle differences between the strains. Furthermore, the anxiety test that showed the largest effects in previous studies (Saoudi et al., 2021), the elevated plus maze, was not included in our study, owing to inaccessibility of the equipment. Interestingly, *DMD-null* mice were more anxious than mice of the other DMD strains, indicating a prominent role of Dp71 and/or Dp40 in anxiety, which has been indicated before in Dp71-null mice, as they are more anxious than WTs (Miranda et al., 2024).

Owing to the lack of Dp260, *mdx52* mice have altered visual processing (Barboni et al., 2021). Although this has not been investigated in *DMD-null* mice, similar deficits could be expected in this model. We cannot rule out that altered visual processing could have influenced the behavior of *mdx52* and/or *DMD-null* mice in these anxiety tests.

Overall, it seems that each of the brain dystrophin isoforms has, to a varying degree, a role in anxiety. Anxious behavior increases with each additional missing dystrophin isoform; however, this change in behavior can be very subtle and difficult to distinguish, especially when only the longer dystrophin isoforms are lacking.

### Freezing phenotype is further exacerbated by the lack of Dp71 and Dp40

The most robust behavioral deficit found in mice lacking Dp427 is the profound freezing response after a short period of restraint (Saoudi et al., 2021; Vaillend and Chaussenot, 2017; Razzoli et al., 2020; Yamamoto et al., 2010; Lindsay and Russell, 2023; Sekiguchi et al., 2009). Our study confirmed that this deficit was present in the *mdx^5cv^* mice and that the additional lack of Dp140 in the *mdx52* mice did not further exacerbate the phenotype. Surprisingly, we observed, for the first time, that *DMD-null* mice show a further increase in freezing compared to *mdx52* mice, and a trend in the same direction was found compared to that in *mdx^5cv^* mice. It should be noted that mice had lost a significant amount of weight in the days prior to the fear test owing to the serial reversal learning task. Although mice recovered quickly and were almost back to their previous body weight before execution of the fear test, a potential influence of this cannot be completely ruled out. Lack of Dp71 and Dp40 seems to have a significant negative effect on the already affected fear response seen in mice lacking Dp427.

### Inconclusive observations for sociability and social novelty seeking

Social interaction has been investigated multiple times in *mdx* mice using the three-chamber social paradigm, with varying results. Although a lack of social preference was reported in 8-week-old *mdx* mice (Alexander et al., 2016), these results could not be replicated in older mice (Miranda et al., 2015; Hashimoto et al., 2022). *Mdx52* mice have been reported to have increased social preference compared to WT and *mdx* mice (Hashimoto et al., 2022). Unfortunately, our setup proved to be suboptimal as WT mice did not show a preference for social interaction or social novelty. Owing to this lack of preference, results on the other groups are inconclusive. We note that, even in the suboptimal setting, *mdx52* mice still showed a small increase in social preference, as described previously (Hashimoto et al., 2022). *DMD-null* mice showed a preference for social novelty in the second trial, but this warrants further investigations.

### Spatial and reversal learning are not decreased by the lack of dystrophin isoforms

Different types of learning and memory were assessed during this study, including hippocampus-dependent spatial learning in relatively low (motor) stress conditions in the Barnes maze and during high (motor) stress conditions in the Morris water maze. Previous studies on *mdx* mice have reported deficits in spatial recall in older (6- and 12-month-old), but not in younger (4-month-old), mice (Remmelink et al., 2016a; Bagdatlioglu et al., 2020; Sesay et al., 1996; Verhaeg et al., 2024). It should be noted that Vaillend et al. (2004) reported impairments in long-term memory in younger *mdx* mice; however, this conclusion was largely based on time spent in the target zone. Owing to the decreased swimming speed of the DMD mouse models, this parameter might not purely represent spatial recall. Reversal learning in the Barnes maze has been reported to be affected in 4-month-old *mdx* mice (Remmelink et al., 2016a). Our results in *mdx^5cv^* mice partly match those in the literature, because we did not find any deficits in spatial learning and recall. We were unable to find the deficits in reversal learning in our *mdx^5cv^* mice that were previously found in *mdx* mice (Remmelink et al., 2016a). Differences in the design of the Barnes maze, with 24 holes in the setup used by Remmelink et al. (2016a), whereas ours only contained 12 holes, could underlie this difference. Furthermore, the difference in the genetic background of the mouse models (*mdx* versus *mdx^5cv^*) could be an underlying factor.

The lack of deficits in *mdx52* mice in acquisition, recall and reversal learning in the Barnes maze is in line with our previous data from *mdx^4cv^* mice, which also lack Dp427 and Dp140 (Verhaeg et al., 2024). Interestingly, the *DMD-null* mice showed improved performance during reversal learning and recall in the Barnes maze. This could be caused by increased motivation to find sheltering, owing to higher anxiety. To our knowledge, no direct correlations have previously been found between anxiety and motivation for shelter seeking in a spatial learning task; however, we noted that WT mice sometimes, after locating the correct hole, did not enter the shelter box, highlighting their lower motivation to seek shelter.

During the higher stress conditions of the Morris water maze, a slight deviation in search pattern was observed in *mdx52* and *DMD-null* mice. These mice did not have trouble reaching the platform location at first; but, after realizing that the platform was absent, they did not show a preference for searching in the quadrant in which the platform should be located, as indicated by the shorter relative distance traveled and time spent in the target quadrant. Possibly, this could be caused by a lower confidence in the platform location, causing them to search more randomly after not finding it. It should be noted that because this effect was only present during the Morris water maze, this is likely an (indirect) effect of the higher motor demand of the task for the animals. Many *DMD-null* mice and several *mdx52* mice had trouble completing the learning day, owing to the numerous acquisition trials. The high motor stress is probably due to the decreased muscle function of these animals (Chesshyre et al., 2022) and their higher body weight.

Learning and memory tasks are highly reliant on the motivation and stress levels of the animal, and this could possibly influence the outcome of the tasks. One should, therefore, always take into account the motivational drive and physical condition of the animals while performing these tests.

### Recognition memory is not affected by the lack of dystrophin isoforms

In terms of recognition memory, the field has been unable to reach consensus about a possible deficit. Whereas some studies found deficits for long-term recognition in the novel object recognition and T-maze tests in *mdx* mice (Vaillend et al., 2004, 1995; Vaillend and Chaussenot, 2017; Comim et al., 2019), others did not (Remmelink et al., 2016a; Bagdatlioglu et al., 2020; Sesay et al., 1996). Dp71-null mice have shown reduced alternation in the T-maze at multiple delays, but did not show any altered behavior in the novel object recognition test (Daoud et al., 2009). Owing to the inconsistencies in the literature, we performed multiple tests, with both short- and long-term delays. We did not observe any deficits in the novel object recognition, object placement or T-maze tests in our current study, although it should be noted that we failed to see preferences in the WT animals in the T-maze and barely saw a preference during the 24 h delay in the object placement test. This could suggest that these protocols were not optimal to study recognition memory, as normally WT mice show a clear preference in these tests (Deacon and Rawlins, 2006; Vogel-Ciernia and Wood, 2014). The lack of preferences in the T-maze makes any conclusions regarding a deficit in the other models inconclusive. Overall, no deficits in recognition memory were observed in the novel object recognition and object placement tests. Results from the T-maze test were inconclusive owing to a suboptimal protocol and possible influences of anxiety on the motivation of the animals.

### Challenges in the food-related reversal task could affect data interpretation

The capacity for flexibility of learning was further examined using a food-rewarded serial reversal task in the automated home cages. *Mdx^5cv^* and *mdx52* mice showed increased performance during the initial discrimination learning. This behavior has been reported in *mdx* mice, although inconsistently (Lewon et al., 2017; Remmelink et al., 2016b; Engelbeen et al., 2021), and is most likely caused by an increased food drive, leading to higher food-seeking behavior (Lewon et al., 2017). Interestingly, *DMD-null* mice did not show this increase in learning. Whether this is due to the lack of an increased food drive or a learning deficit remains unclear. It should, however, be noted that a preference for the left hole could not be ruled out as the target hole was not randomized between individuals. After the initial reversal learning switch, *mdx^5cv^* mice showed a decrease in performance compared to that of WT mice; this decrease in performance was borderline in *mdx52* mice and absent in the *DMD-null* mice. The participation of *mdx52* and *DMD-null* mice was noticeably lower during the first reversal day, indicative of a lower engagement overall, which could result from anxiety (Fig. S8). It remains unclear why activity was most heavily affected during this specific day. Interestingly, the lack of activity in *mdx52* and *DMD-null* mice and the probable lack of establishment of this new location seemed to have a bigger impact on *mdx52* mice, as only these mice showed improved performance during the second reversal trial, when the target location goes back to the initially learned location. This was confirmed by the perseverative error curve in which *mdx52* mice already start at a much lower fraction, showing less interest in the previous target than the other groups. A potential preference for the left hole in the discrimination trial in some strains could also be an underlying cause for the altered performance in the first and second reversal trials.

During the next reversal day, in which the middle hole was introduced as a target, all DMD models outperformed the WT mice, which had a lower affinity for the target hole than for both lateral holes. We hypothesize that the WT mice learned a stronger correlation or pattern during the first days in the PhenoTyper cages, where they specifically correlated both lateral entrances with food rewards, whereas the different DMD mice did not recognize this left-right-left pattern. Therefore, it would be easier for the DMD mouse models to adjust their behavior, because their switches would not favor the lateral sides necessarily. Taken together, these results suggest that *DMD-null* mice behave differently to the other DMD models in this food-motivated reversal task, possibly owing to lack of increased discrimination learning and lack of decreased initial reversal learning. Additionally, problems with pattern recognition in all DMD models could be present and should be investigated further. However, as many factors can influence behavior in the reversal learning trials, including food motivation, progressive impact of the prolonged food restriction, preference for a specific hole, confusion induced by previous trials and overall activity, conclusions should be made tentatively.

### Lack of Dp140 results in lack of habituation; loss of Dp71 and Dp40 lead to restlessness

One of the great advantages of the tasks performed in the PhenoTyper cages is that there is minimal disruption from unintentional external stimuli, which is especially important when doing deep phenotyping analysis of spontaneous behavior. It should, however, be noted that the single housing conditions could impose stress, especially in DMD mice, which are known to be sensitive to stress, thereby potentially influencing results. Overall, we saw many subtle differences in activity-based behavior in the *mdx52* and *DMD-null* mice. The deviations in *mdx52* behavior were more inconsistent and subtle, and mostly related to changes from the dark to light phase. Notably, *mdx52* mice also showed a lack of habituation, which could have an impact on the execution of other behavioral tests, such as learning tasks, which rely on habituation to decrease anxiety effects. It would be beneficial in future studies to prolong the experiments to investigate how long this lack of habituation remains present. Previous experiments in *mdx* and *mdx^4cv^* mice, lacking Dp427 and Dp427+Dp140, respectively, have shown deviations in spontaneous behavior in similar directions in most, but not all, parameters (Verhaeg et al., 2024). It should be noted that the duration of the experiment differed between the studies [2 days in Verhaeg et al. (2024) versus 3 days in this study]. Habituation can play an important role in behavioral changes; this could have contributed to the differences between the studies. Also, the use of different strains [*mdxbl6* versus *mdx^5cv^* and *mdx^4cv^* versus *mdx52* in Verhaeg et al. (2024)], could have played a role. Lastly, the strain of the subsequent testing could have influenced behavior in this study, especially in *mdx52* and *DMD-null* mice, as they showed difficulties in completing the Morris water maze test, which was performed 24 h before the start of the PhenoTyper analysis.

The *DMD-null* mice showed overall shorter activity bouts, less reactivity to dark and light changes, decreased duration of long shelter visits, and shorter movement and arrest bouts compared to WT mice. This suggests that the activity of the *DMD-null* mice is more erratic, having more alternations between activity and rest, without necessarily affecting the total amount of activity. This restless type of behavior has been observed previously (Kudoh et al., 2005) but had not been quantified until now. Furthermore, the decreased time of long shelter visits could indicate a disruptive sleep pattern, as these long shelter visits are usually multiple hours long. Taken together, the *DMD-null* mice appear to be more restless in their spontaneous behavior, which is illustrated by multiple outcome measures.

### Translational value

Although the severity and abundance of alterations – such as anxiety, fear and reversal learning – seen in *DMD-null* mice are partly in line with those made in patients, it should be noted that the differences found between mice lacking Dp427 and Dp140 versus mice also lacking Dp71 and Dp40 seem to be less substantial and abundant than the differences seen in corresponding patients, in whom the additional lack of Dp71 and Dp40 leads to a very strong phenotype (Taylor et al., 2010; Daoud et al., 2009; Moizard et al., 2000; Lenk et al., 1993). Further analysis of this model and its translational value is warranted. Possibly, the differences observed between clinical and preclinical data could be influenced by the lack of corticosteroid administration in our mouse models. Corticosteroids are part of the standard of care in DMD and used by the vast majority of patients to slow down muscle degeneration (Khan, 1993; Fenichel et al., 1991). Corticosteroids are known to have negative effects on behavior in healthy humans and mice (Prado and Crowe, 2019; Schmidt et al., 1999; Ciriaco et al., 2013; Kajiyama et al., 2010; Dos Santos et al., 2019). The effects of corticosteroids on behavior in patients with DMD and mouse models remain largely unclear; however, it is known that the treatment regime of corticosteroids is linked to brain pathology, gray matter volume, specifically, in patients with DMD (Geuens et al., 2023), and depressive-like behavior in *mdx* mice (Liu et al., 2024). We cannot therefore rule out that the lack of corticosteroid treatment in this study might have contributed to the differences seen between our mouse models and patients with DMD.

### Limitations

Although this study has provided new insights into the deficits of DMD models, the study design used could have (partly) influenced outcomes. First, mice were housed in groups of two to four mice in individually ventilated cages. The variation in social environment and the use of ventilated cages could both have influenced behavior such as depression and anxiety, and make comparisons to the literature more complicated (York et al., 2012; Horii et al., 2017). Other types of behavior, such as recognition memory and social interaction, seem to be unaltered by housing in individually ventilated cages.

Second, to minimize the amount of animals required for the study, we chose an elaborate setup of subsequent behavioral testing, meaning that mice were subjected to several tests in a short time period. Although the order of testing was chosen such as to minimize stress of the cumulative testing, we cannot rule out that results were influenced by this study design. To minimize this risk, tests inducing high stress levels, such as the Morris water maze, were conducted at the end of the test battery, and data analysis of the subsequent PhenoTyper cage test was restricted to the third night they spent there. It cannot be ruled out that spontaneous behavior was affected by this approach.

### Conclusions

This study aimed for a broad generic behavioral description and deep phenotyping of mice lacking one, multiple or all brain dystrophin isoforms. We confirmed already-established deficits of anxiety and fear in *mdx^5cv^* and *mdx52* mice and showed that this was further exacerbated in *DMD-null* mice, indicating a specific role of Dp71/Dp40 in anxiety and fear responses. We showed that *mdx52* and *DMD-null* mice suffer from subtle deficits in spatial memory only when under high motor stress. Lastly, we showed subtle changes in spontaneous behavior, especially in *mdx52* and *DMD-null* mice. Altogether, this study provides the field with an extensive overview of behavioral deficits in different DMD models, giving new insights into the behaviors in which dystrophin isoforms are involved, which could be used in future preclinical research.

## MATERIALS AND METHODS

### Mice

Male *mdx^5cv^* (B6Ros.Cg-*Dmd^mdx^*^-5Cv^/J) (Im et al., 1996), *mdx52* (Araki et al., 1997), *DMD-null* (Kudoh et al., 2005) and WT mice, all on a C57BL/6J genetic background, were bred at the animal facility of the Leiden University Medical Center. For all strains, heterozygous females were paired with WT males to produce DMD and WT male littermates. WTs from all three strains were pooled in one group, which consisted of eight *mdx^5cv^* WT, eight *mdx52* WT and 16 *DMD-null* WT males. Other groups all consisted of 16 males. Mice were genotyped after birth and at the end of the study (Fig. S1). One animal was mislabeled at the start of the study, resulting in nine *mdx52* WT mice and 15 *mdx52* mice. Mice were housed in groups of two to four animals in individually ventilated cages (Makrolon type II, Tecniplast, London, UK) filled with sawdust and enriched with nesting (Bed-r'Nest BRN8SR, Life Science Equipment, Oud-Turnhout, Belgium) and bedding (LIGNOCEL BK-8-15-00433, JNR, Holzmühle, Germany) materials and a cardboard tunnel (GLP Fun Tunnels Mini, 1022006, LBS, Surrey, UK). *Ad libitum* access to standard RM3 chow (SDS, Essex, UK) was provided to the mice, except during the food reward task in the PhenoTyper cages. *Ad libitum* access to water was provided to the mice during the whole study. Mice were exposed to a 12h:12 h, dark-light cycle, with lights being turned on between 07:00 and 19:00. All tests were performed in rooms dedicated to behavioral experiments during the light phase between 07.00 and 17.00, with the exception of the continuous tracking in the PhenoTyper cages. Timing of each test was kept as consistent as possible for all the animals, meaning that although some tests were performed in the morning and others in the afternoon, tests started at roughly the same time of the day for all cohorts. Researchers were unaware of the genotypes while performing the tests. Tests were primarily performed by three female researchers. Animals were handled by their tail when placed into an experimental setting.

Experiments were approved by the Animal Ethics Committee of the Leiden University Medical Center (AVD 1160020171407, PE.17.246.037) and executed conforming to Directive 2010/63/EU of the European Parliament. The animal study was also reviewed and approved by the Central Authority for Scientific Procedures on Animals and performed according to Dutch regulations for animal experimentation.

### Experimental setup

Mice entered the study at 8 weeks of age and underwent multiple behavioral tests, while body weight was recorded on a weekly basis (Fig. S2). Tests included the dark-light box, open field, three-chamber social interaction, Barnes maze, T-maze, novel object recognition, object placement, Morris water maze and restrained unconditioned fear tests. At 14 weeks of age, mice were housed in PhenoTyper automated home cages (model 3000, Noldus Information Technology, Wageningen, The Netherlands), in which spontaneous behavior, discrimination learning and reversal learning were also assessed (Fig. 1). At least 1 day of rest was provided between each of the tests. Between trials, materials were cleaned with 70% ethanol. Behavior was tracked automatically using Ethovision XT (Noldus Information Technology), at a rate of 20 frames/s for the dark-light box, open field, Morris water maze and unconditioned fear tests, and in the PhenoTyper cages. Interactions with objects in the three-chamber social interaction, Barnes maze, novel object recognition and object placement tasks were scored via automated tracking using DeepLabCut (Nath et al., 2019; Mathis et al., 2018), instead of Ethovision, owing to difficulties in tracking of the partially obscured animals caused by the camera angle and position of the objects. In short, DeepLabCut was trained on ±1000 labeled frames to detect the nose, head, ears, shoulders, back and tail of the mice. Coordinates of the locations of the body parts were extracted per frame, and the location of the objects or target holes was determined with in-house image segmentation routines. Interaction was described as being in close proximity to the object or hole. Scripts were developed for each test separately and validated on separate datasets with manually scored data from two independent scorers [correlation scores varied between 0.80 and 0.98 for the different behavioral tests]. All testing equipment was made in-house, with the exception of the PhenoTyper cages. At 17 weeks of age, mice were sacrificed using CO_2_.

### Behavioral tests

#### Dark-light box

Anxiety was measured with the dark-light box, which consisted of two compartments (50×25 cm each) connected via a door (10×5 cm) (Saoudi et al., 2021). Animals were placed in the dark compartment, and, after 20 s, the door was opened, and animals were allowed to freely explore both compartments for 5 min. Behavior was only recorded in the light compartment.

#### Open field test

To further assess anxiety, mice were exposed to the open field test. Animals were released into a white box (50×50×35 cm) for 30 min. The box was digitally divided into an inner zone (region of 40×40 cm in the middle of the box) and outer zone (the remaining area) during analyses. Location and movement of the mice were measured.

#### Three-chamber social interaction test

The three-chamber social interaction test was used to investigate social interaction in a controlled environment (Miranda et al., 2015). The setup consisted of three chambers (21×42×35 cm each), which were connected by doors (10×5 cm). The outer walls of the box were opaque, and the inner walls dividing the chambers were transparent. A black tube (height, 20 cm; diameter, 8 cm; metal bars spaced 1 cm apart) was placed in each lateral chamber, in such a way that the center of the tube was placed 10 cm from the outer walls. Mice used for the interaction (strangers) were all male WT mice. In total, six pairs of two strangers were used to keep age differences between strangers and experimental mice at a minimum (maximum age difference was 2 weeks). During the habituation trial, the experimental mouse was placed in the middle chamber, and each lateral chamber contained an empty black tube. After 2 min, the doors to the lateral chambers were opened, and the mouse was recorded for 10 min. In between trials, the experimental mouse was placed back in the home cage shortly to allow cleaning of the equipment. During the first trial, which was performed almost directly after habituation, an object was placed into one of the tubes. The other tube contained a novel mouse (stranger 1), which was unfamiliar to the experimental mouse. At the start of the trial, the experimental mouse was placed in the middle chamber again, and doors were opened after 30 s. The mouse could walk around freely for 10 min. During the second trial, which was performed directly after the first trial, stranger 1 was placed in a tube in the same lateral chamber as in the previous trial. The other tube contained a new mouse (stranger 2), which was again unfamiliar to the experimental mouse. The experimental mouse was placed in the middle chamber again. After 30 s, the doors were opened, and the mouse could walk around freely for 10 min. Interaction with the objects and strangers was measured and quantified. DIs were calculated for sociability [trial 1: interaction time stranger 1/(interaction time stranger 1+interaction time object)] and for social novelty seeking [trial 2: interaction time stranger 2/(interaction time stranger 1+interaction time stranger 2)].

#### Barnes maze

Spatial learning and memory were assessed with the Barnes maze, which was a circular platform (120 cm diameter) made from wood covered with a water-resistant top, which included 12 holes (10.5 cm diameter) equally spaced apart 12 cm from the circumference of the maze (Remmelink et al., 2016a). A transparent platform beneath one of the holes (target hole) led to a hidden escape box. Visual cues were positioned around the maze for spatial orientation. During the first 5 days, two acquisition trials were held daily, with a 5 min interval between trials. During each trial, mice were placed in the middle of the maze and could try to locate the hidden platform for 5 min. If mice failed to reach the platform during this timeframe, they were manually placed into the correct hole. Mice could stay in the escape box for 30 s before being taken out. During the second trial of day 5, the platform was removed, and behavior was recorded for 5 min. No trials were performed on day 6 and 7. On day 8, two more learning trials were held in the same manner as described before, with the escape box in place. On day 9 and 10, the platform and escape box were transferred to the opposite side of the maze. Three learning trials took place on day 9, with the same setup and interval as described before. On day 10, one learning trial similar to those of day 9 took place, but, during the second trial, the platform was removed again. Distance to target hole and interaction with all the holes were measured as an indication of spatial learning and memory.

#### T-maze

Recognition memory was tested with the T-maze (Vaillend et al., 1995). The T-maze consisted of one start arm (35×10 cm), including a start box (13×10 cm) and two lateral arms (35×10 cm). Mice were placed in the start box at the start of each trial. On the first day (habituation trial), the start box and both lateral arms were open, and the mice could explore the maze freely for 5 min. During day 2 and 3, mice were kept in the start box for 30 s before the latch was lifted. On day 2, two consecutive learning trials were held, during which one lateral arm was closed off. The trial ended 30 s after the mice entered the open lateral arm. During this time, mice were not restricted to the lateral arm and allowed to walk back to the start arm. All mice entered the lateral arm freely within 5 min. In between the trials, mice were briefly placed in their home cage to allow cleaning of the apparatus. After a 24 h delay (test trial), both arms were open again, and the mice were allowed to freely explore the maze for 5 min. After 6 days of rest, learning and test trials were repeated, with a delay of 6 h between the last learning trial and the test trial. Choice of first entry (alternation) and total time spent in each arm were measured to assess recognition memory. DIs were calculated to analyze preference for the novel arm: time spent in novel arm/(time spent in novel+familiar arm).

#### Novel object recognition

Recognition memory was further investigated using the novel object recognition test. The open field box was filled with bedding, and objects of different shapes, colors and textures were selected for equal exploration. Objects were held in place by magnets on the exterior of the box and placed ∼10 cm from the walls. Initially, 12 objects were tested for spontaneous interaction with eight WT mice (mice were exclusively used to assess suitability of the objects), meaning that the WT mice were subjected to sets of three random objects at a time for 10 min and interaction times were measured. Two objects that evoked significantly higher interaction compared to that for the other objects were excluded from further use.

Four days of habituation preceded the assessment of recognition memory (Vaillend et al., 2004). On the first day, two habituation sessions, spaced at least 4 h apart, took place, during which all mice from one cage were placed in the box together for 10 min. On day 2 and 3, the mice were individually placed in the box to explore for a single 10 min session. This was also done on day 4, but three dedicated objects, which were not used for the remainder of the experiment, were placed in the box. Learning trials were started 2 days after the last habituation session. Mice were individually placed in the box with three objects for three 5 min trials with 5 min intervals between them. After ∼24 h, a 5 min test trial was performed in which one of the three objects was replaced by a novel object. After a maximum of 2 resting days, learning and test trials were repeated with a set of three novel objects in which a 10 min delay instead of a 24 h delay was used between learning and test trials. Objects were pseudo-randomly selected for each mouse, meaning that object combinations and locations of the novel object (left, middle or right position) were similar between groups. Bedding was not changed for the duration of the experiment for one cohort (maximum of eight mice). The interaction time with each object was measured, and DIs were calculated during the test trials: interaction time novel object/(interaction time novel+old objects).

#### Novel object placement

The novel object placement task was performed to assess spatial memory. The test was also done in the open field box, utilizing the same bedding as for the novel object recognition test. Distinct black visual cues were added to the walls of the box for spatial orientation. Two identical objects were pseudo-randomly placed in the box; objects and relative locations (next to each other versus in opposite corners) were similar between groups.

The mice were placed in the middle of the box, and three learning trials were conducted for 5 min with a 5 min interval between them. During the test trial, 24 h later, the location of one of the two objects was pseudo-randomly changed. After a maximum of 2 days of rest, learning and test trials were repeated with a 10 min interval between the last learning trial and the test trial. The interaction time with the objects was measured and DIs were calculated: interaction time novel object location/(interaction time novel object location+old object location).

#### Morris water maze

The Morris water maze was used to assess spatial memory further (Vaillend et al., 2004). A round bath (120 cm diameter) was filled with water (at 26°C) and white dye, making it opaque to prevent mice from visually navigating to a hidden platform (11 cm diameter) just beneath the water surface, located ∼27 cm from the edge of the pool. Visual cues were placed around the maze for spatial orientation. Four quadrants were determined in the maze: north-west (NW), south-west (SW), north-east (NE) and south-east (SE), with the platform positioned in the NE quadrant. Mice were habituated for 1 day during two sessions, with a minimum interval of 4 h. Each session consisted of four consecutive trials, during which the mice were guided by hand through the water towards the platform, on which they could stay for 60 s. If mice jumped off the platform, they would swim around freely until the 60 s were over. On the second day, the platform location was acquired during five sessions, with a 15-20 min interval between the sessions. Each session consisted of five consecutive trials. Mice were placed pseudo-randomly in one of the quadrants at the beginning of each trial (with the exception of the target quadrant that contained the platform). Trials lasted for 1 min or until the mice found the platform, after which they could stay on the platform for 2 min. If the platform was not found after 1 min, mice were put on the platform by hand. Spatial memory was assessed 24 h later via a probe trial in which the platform was removed. Mice were pseudo-randomly placed in one of the quadrants (with the exception of the target quadrant that had previously contained the platform) and could freely swim for 1 min. They were allowed to warm up under a heating lamp for ∼15 min after each session. The protocol was stopped prematurely if the mice had trouble keeping their head above the water surface, which was the case for two *mdx52* and five *DMD-null* mice. Distance swam until finding the platform or entering the platform zone was measured during each trial. Additionally, swimming speed and time and distance in each quadrant were analyzed.

#### PhenoTyper automated home cages

PhenoTyper automated home cages (model 3000, Noldus Information Technology) consist of transparent Perspex walls (30×30×35 cm) and opaque Perspex floors and an infra-red camera in the top unit. Drinking and feeding stations were included in the cage, as well as a rectangular shelter (10×10×5 cm) with two entrances (3 cm diameter). Cages were filled with sawdust bedding during all experiments.

#### Spontaneous behavior

To assess spontaneous behavior, mice were tracked continuously for 3 days with minimal interference (Loos et al., 2014). Mice were put in the PhenoTyper cages during the light phase (between 13:00 and 16:00). Spontaneous behavior was analyzed starting at the beginning of the upcoming dark phase (19:00). Mice were individually housed and had *ad libitum* access to standard RM3 chow and water. Spontaneous behavior was defined with 20 previously described parameters (Loos et al., 2014) for the dark and light phase of day 3.

#### Discrimination and reversal learning task

The day after spontaneous behavior analyses were completed, cognitive flexibility was assessed via a food-rewarded discrimination and reversal learning task. Standard chow was removed, and a cognition wall (17 cm wide, 25 cm high) with three circular entrances (3 cm diameter) was introduced in the corner opposite the shelter. A pellet dispenser, dispensing food pellets (Dustless Precision Pellets, 14 mg, Rodent Purified Diet, Bio-Serv, Frenchtown, NJ, USA), was placed such that the dispenser tube protruded behind the cognition wall. A pellet was released after each fifth correct entry through the target entrance. Correct entries did not have to be consecutive. Discrimination learning lasted for 48 h, during which the left hole was deemed the target hole. During the remaining 5 reversal days, the target entry was switched daily in the following pattern: right, left, middle, right, left.

Six *mdx^5cv^*, one *mdx52* and one *DMD-null* mice were excluded from analysis owing to technical issues during the test. Animals were weighed daily (Fig. S2B) and given extra food if weight loss was more than 2 g (∼7% weight loss) in 24 h or more than 5 g since the start of the task (∼15-20% weight loss). In total, one WT, one *mdx^5cv^* and five *DMD-null* mice were taken out of the task prematurely [mostly between reversal learning day (RL)1 and RL2] owing to extreme weight loss or signs of inactivity and distress. Learning abilities were assessed by calculating the amount of correct entries during a 50-entry window. After the test, animals were individually housed in individually ventilated cages, to prevent fighting after regrouping of adult males. They had *ad libitum* access to water and standard RM3 chow for the remainder of the study (2 days).

#### Restrained unconditioned fear test

To examine fear response without the interference of fear learning or association learning, freezing behavior was assessed after evoking an unconditioned fear response (Saoudi et al., 2021). Mice were held upside down for 15 s by scruffing the neck. Afterwards, they were released into the open field box, and freezing behavior was tracked for 10 min.

### Genotyping

Ear cuts were made for identification and genotyping prior to weaning and at the end of the experiment after sacrifice to reconfirm the genotype of all mice. Ear pieces were suspended in a 50 µl buffer made of 100 mM Tris, 5 mM EDTA, 200 mM NaCl, 0.2% SDS to which 0.5 µl Proteinase K (Roche Diagnostics, Mannheim, Germany) was added, and samples were incubated overnight at 55°C in a shaking water bath. Proteinase K was inactivated for 10 min at 97°C, and samples were spun down for 30 s at 10.25 ***g***. Then, 1 µl DNA was added to the PCR mix [0.4 µl dNTPs, 4 µl 5× Phire Reaction buffer (Thermo Fisher Scientific, Waltham, MA, USA), 1 µl forward primer (10 pmol), 1 µl reverse primer (10 pmol), 0.2 µl Phire Hot Start II DNA Polymerase (Thermo Fisher Scientific) and 12.4 µl H_2_O]. The following PCR program was used: 30 s at 98°C, followed by 30-35 cycles of 5 s at 98°C, 5 s at a primer-specific annealing temperature (Table S1), 10 s at 72°C. After the repeated cycles, the PCR was ended with 1 min incubation at 72°C. For the *mdx^5cv^* samples, a restriction endonuclease digest by the DraIII enzyme was performed. For this, 5 µl digestion mix [CutSmart Buffer 10×, Drall-HF, New England Biolabs, Hitchin, UK (20 U/µl), dH_2_O] was added to 10 µl PCR product and incubated at 37°C for 45 min. Samples were run on a 2-3% agarose gel (Fig. S1). PCRs were performed in duplicate for each sample.

### Data analysis

Statistical tests were performed in SPSS and RStudio. Data were assessed for normality and log10 transformed if needed to achieve normality (this was required for the Barnes maze distance walked in the reversal probe and the Morris water maze time in NW, SE and SW quadrants). If normality was confirmed, a one-way ANOVA test with Tukey post-hoc was performed to compare group differences. Comparisons to chance level were done with a one-sample *t*-test. If data were not normal, group differences were tested with the Kruskal–Wallis test, and, if significance was found, repeated Mann–Whitney tests were performed to further analyze group differences. To compare chance levels in data that were not normally distributed, the one-sample Wilcoxon signed rank test was performed per group. Changes in behavior over time were assessed using linear mixed models in RStudio (version 4.3.1) via the LmerTest package (version 3.1.3). Group differences in terms of alterations in the T-maze were tested with a chi-square test. Because differences between WT groups were minimal (Table S2), WTs were pooled into one group, except for the body weight analysis. DMD models were tested for significant differences against this combined WT group, and direct comparisons between DMD models were only done if at least one of the DMD models showed a significant difference compared to WTs. *P*<0.05 was considered significant. Spontaneous behavior data were corrected using false discovery rate via the False Discovery Rate Online Calculator. Graphs were made via GraphPad, RStudio and Adobe Illustrator. All data are shown as mean±s.e.m. Additional statistical information can be found in Tables S2 and S3.

## Supplementary Material

10.1242/dmm.052047_sup1 Supplementary information
