# Supplementary material for: The behavioural consequences of dystrophinopathy
Source: Dis Model Mech. 2025 Mar 3;18(2):DMM052047. doi: 10.1242/dmm.052047 (PMC11911635; doi:10.1242/dmm.052047)
Supplement: Supplementary information [file dmm-18-052047-s1.pdf]

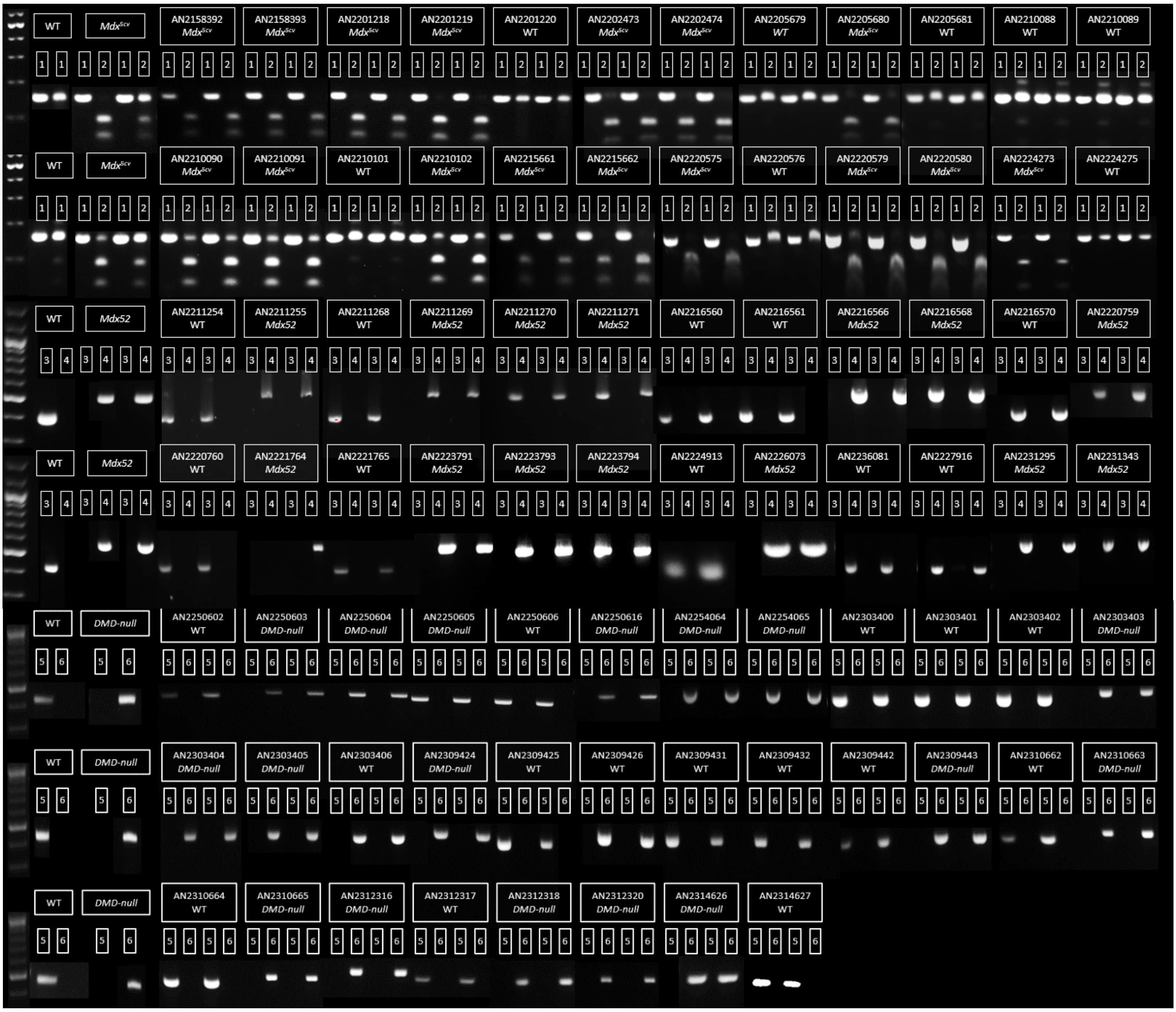

Fig. S1. Genotyping blots of DMD mouse models. Genotyping performed after birth. Numbers correspond to primer pairs as described in Table S1. Product size per primer pair; primer 1: 147 bp (present in *mdx*^{5cv} and corresponding WT samples), primer 2: 93 bp & 54 bp (present in *mdx*^{5cv} samples), primer 2: 383 bp (present in corresponding WT samples), primer 4: 493 bp (present in *mdx52* samples), primer 5: 437 bp (present in corresponding WT samples), primer 6: 453 bp (present in *DMD-null* samples).

Disease Models & Mechanisms • Supplementary information

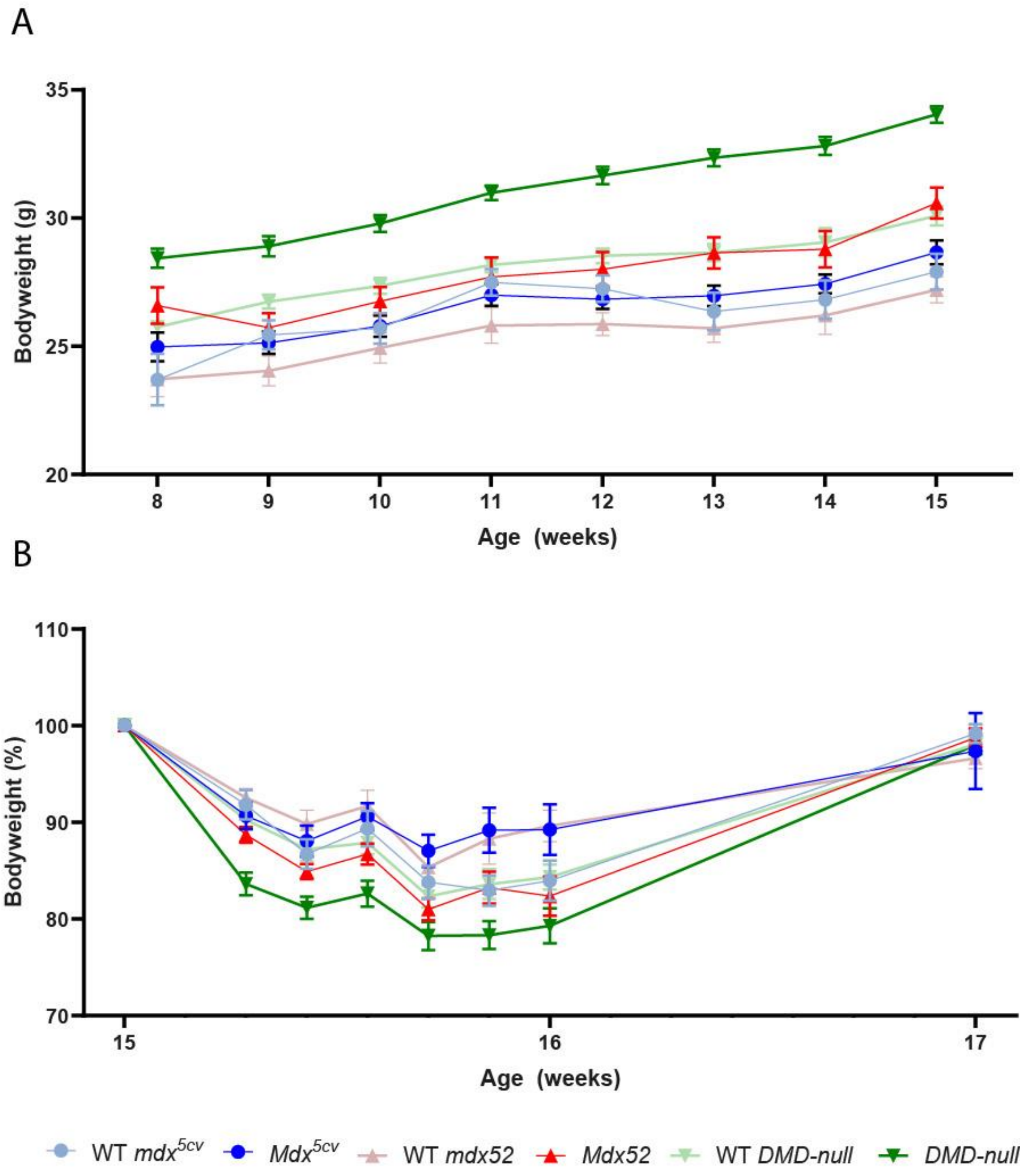

Fig. S2. Bodyweight through the study. *Mdx*^{5cv} (n = 16), *mdx52* (n = 15), *DMD-null* (n = 16) and WT mice (n = 33). A) Bodyweight of *mdx52* and *DMD-null* mice was increased compared to their respective WT groups (P = 0.017 and P < 0.001). No direct comparisons were made between DMD models due to differences between WT groups (P < 0.001). B) Bodyweight during spontaneous behavior and serial reversal learning in the PhenoTyper cages, as a percentage of the weight measured before starting the discrimination task. No statistical tests were performed.

Disease Models & Mechanisms • Supplementary information

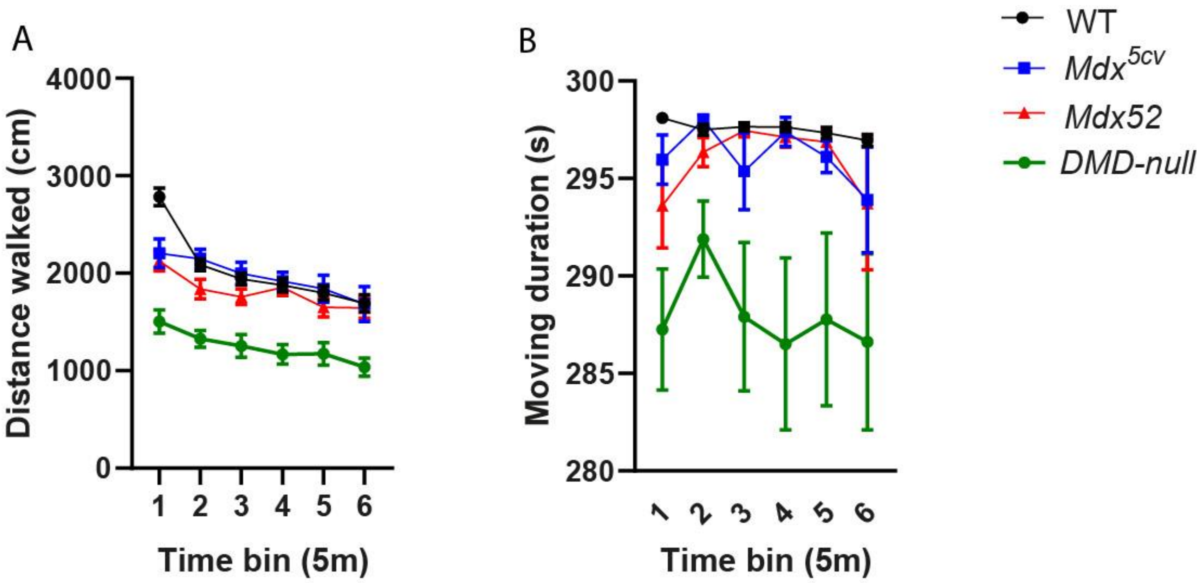

Fig. S3. Locomotion in the open field task. *Mdx*^{5cv} (n = 16), *mdx52* (n = 15), *DMD-null* (n = 16) and WT mice (n = 33). A) The initial longer distance walked in the first time bin for WT mice was not seen in *mdx*^{5cv}, *mdx52* or *DMD-null* mice (P = 0.04, P = 0.005 and P = 0.002 respectively). Overall, *DMD-null* mice walked a shorter distance compared to the other strains but no differences in distance walked over time could be found between the DMD strains. B) While *DMD-null* mice spent less time moving overall, no difference in moving pattern could be detected between the different strains.

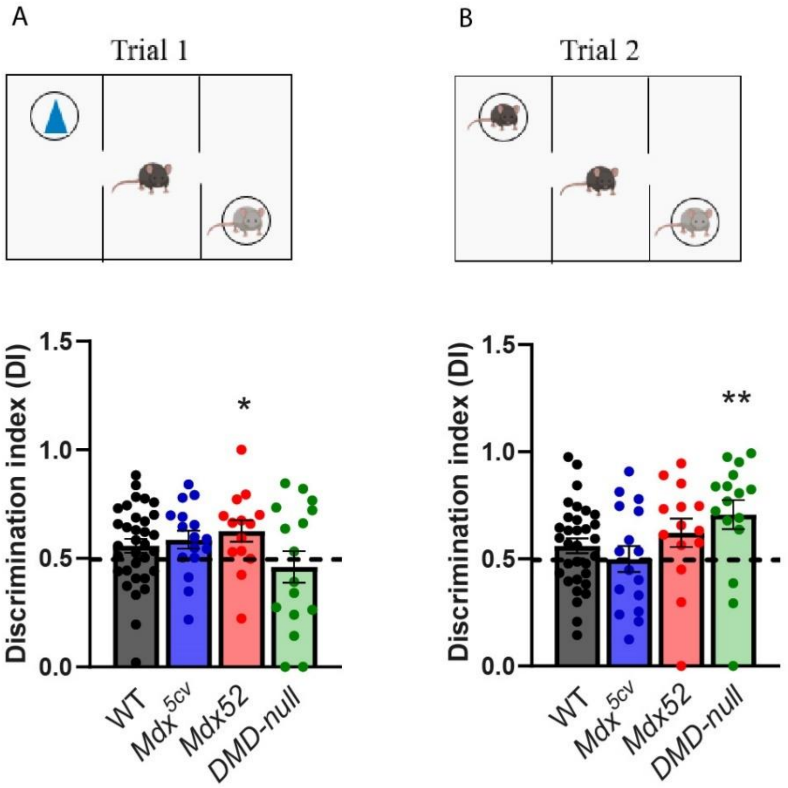

Fig. S4. 3 chamber social interaction test. *Mdx*^{5cv} (n = 16), *mdx52* (n = 15), *DMD-null* (n = 16) and WT mice (n = 33). A) WT mice did not show a significant discrimination index (above chance level). No differences were found between groups in social preference. *Mdx52* mice showed a preference towards social interaction over object interaction when compared to chance level (P = 0.024), but no conclusions can be drawn from this. B) Again, no performance above chance level was observed for the WT mice. No differences were found between groups in terms of social novelty seeking. *DMD-null* mice did show a preference towards the novel social interaction compared to familiar social interaction, when compared to chance level (P = 0.008). Dashed lines represent the chance level. Cartoons were created with BioRender. Created in BioRender by Verhaeg, M. (2025). https://BioRender.com/w29h177. This figure was sublicensed under CC-BY 4.0 terms. *: $P < 0.05$, **: $P < 0.01$.

Disease Models & Mechanisms • Supplementary information

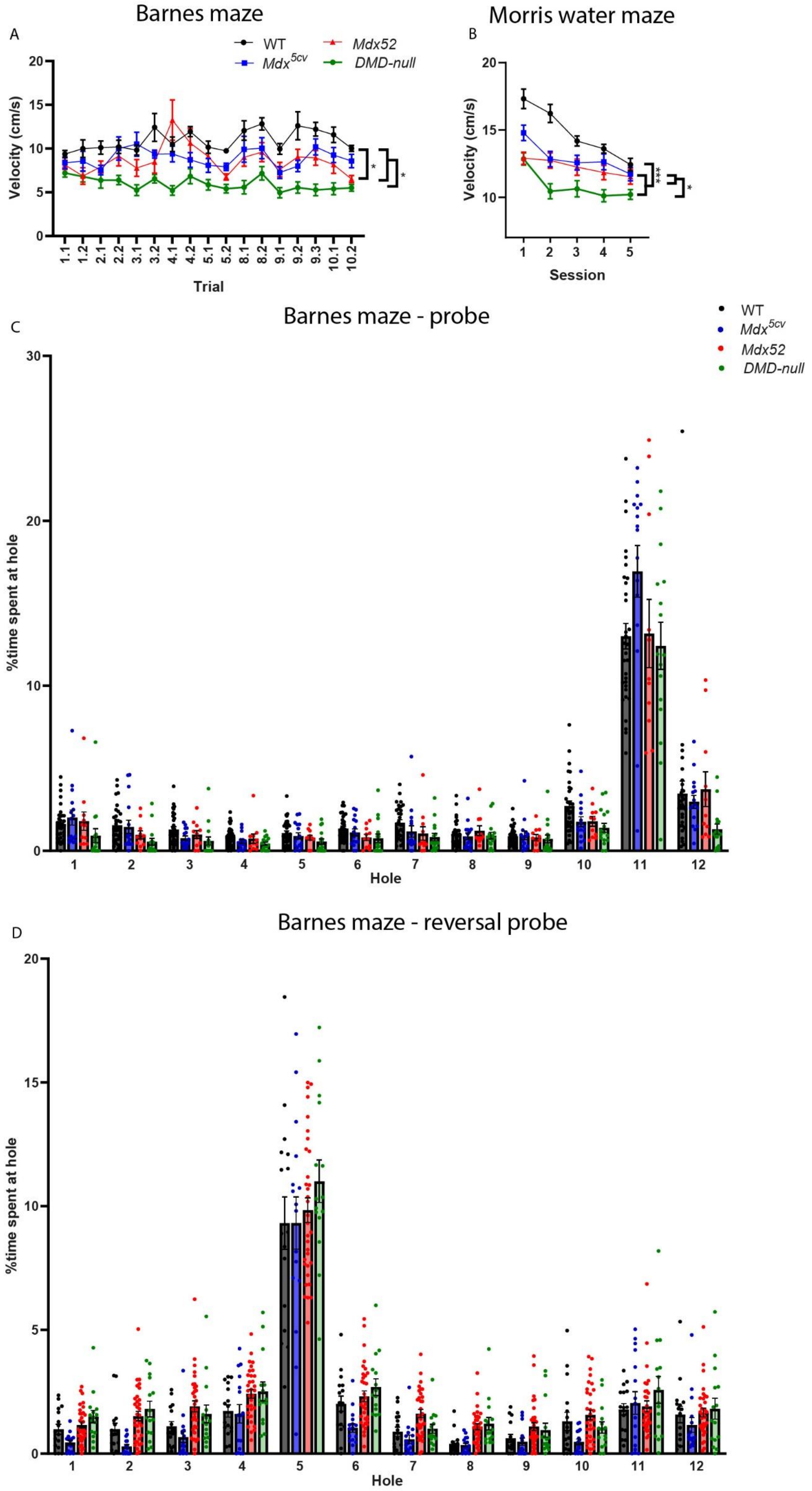

Disease Models & Mechanisms • Supplementary information

Fig. S5. Morris water maze and Barnes maze velocity and interaction time. *Mdx*^{5cv} (n = 16), *mdx52* (n = 13-15), *DMD-null* (n = 11-16) and WT mice (n = 33). A) While navigating the Barnes maze, *DMD-null* mice walked slower compared to WT, *mdx*^{5cv} and *mdx52* mice (P < 0.001, P = 0.010 & P = 0.019 respectively). *Mdx52* mice displayed a lower walking velocity compared to WTs (P = 0.045). B) In the Morris water maze, *mdx*^{5cv}, *mdx52* and *DMD-null* mice all swam slower compared to WT mice (all P < 0.001). *DMD-null* mice also swam slower than *mdx*^{5cv} and *mdx52* mice (P < 0.001 & P = 0.017 respectively). C-D) Percentage of time spent at each hole was calculated for probe (C) and reversal probe trials (D). No differences were found between groups in interaction times with the target hole during the probe (hole 11) and reversal probe (hole 5) trial of the Barnes maze test. *: P < 0.05, ***: P < 0.001.

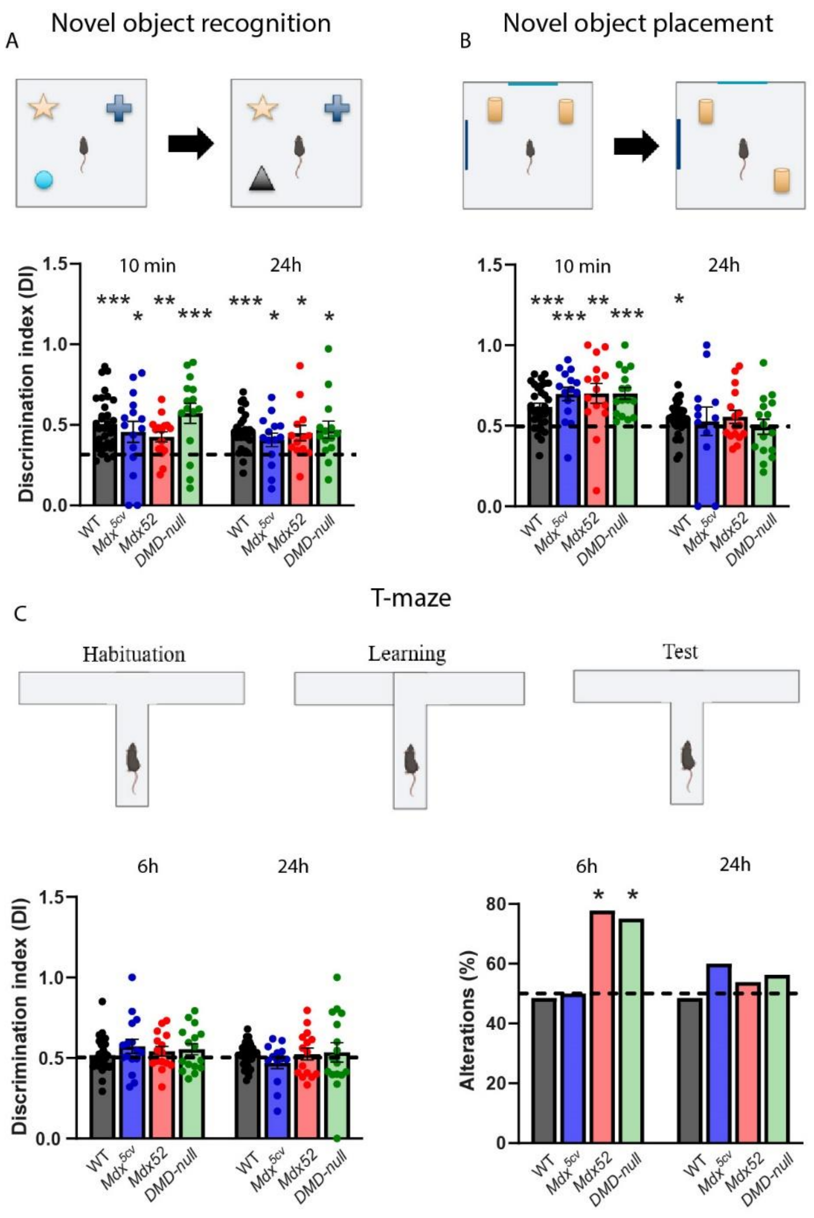

Fig. S6. Recognition memory during the novel object recognition task, the object placement task and the T-maze.

Mdx^{5cv} (n = 16), *mdx52* (n = 15), *DMD-null* (n = 16) and WT mice (n = 33). A) Discrimination index of the novel object recognition task did not differ between groups at either the 10 minute or 24h delay. Almost all groups performed above chance level (0.33) during the 10 minute (WT: $P < 0.001$, *mdx*^{5cv}: $P = 0.036$, *mdx52*: $P = 0.004$, *DMD-null*: $P < 0.001$) and 24h delays (WT: $P < 0.001$, *mdx*^{5cv}: $P = 0.044$, *mdx52*: $P = 0.015$, *DMD-null*: $P = 0.011$). B) No differences found between groups for the DI during the object placement task at either the 10 minute or 24h delay. All mice performed above chance level (0.5) after the 10 minute delay (WT: $P < 0.001$, *mdx*^{5cv}: $P < 0.001$, *mdx52*: $P = 0.003$, *DMD-null*: $P < 0.001$). Only WT mice performed above chance level after the 24h delay ($P = 0.014$). C) No differences were found between the groups at either the 6h or 24h delay in the T-maze in terms of DI values or alternations. None of the groups differed from chance level (0.5). *Mdx52* and *DMD-null* mice alternation levels were above chance level ($P = 0.02$ & $P = 0.046$ respectively). Dashed lines depict the chance level. Cartoons were created with BioRender. Created in BioRender by Verhaeg, M. (2025). https://BioRender.com/v90d559. This figure was sublicensed under CC-BY 4.0 terms. *: $P < 0.05$, **: $P < 0.01$ ***: $P < 0.001$.

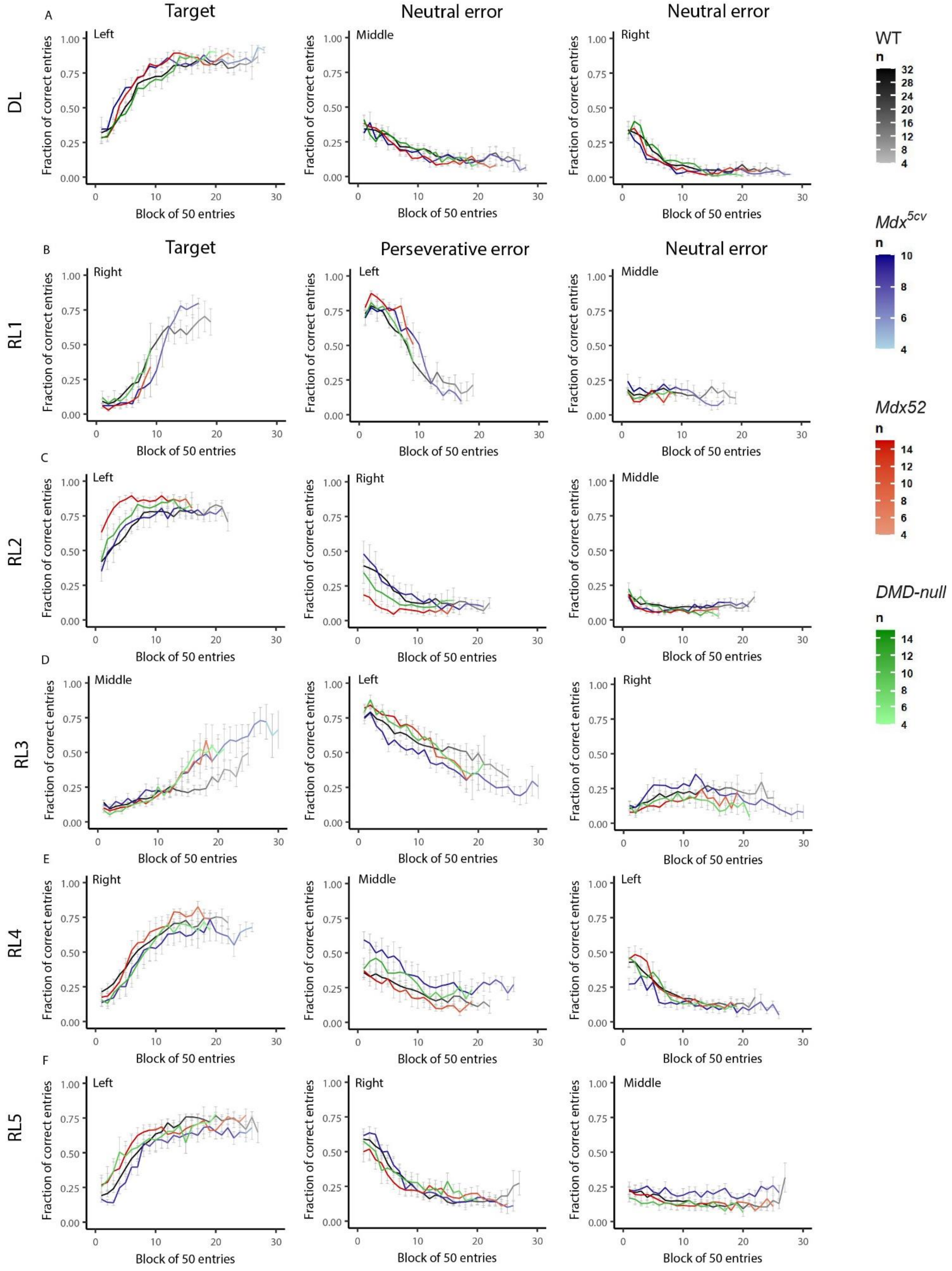
A
Target
Neutral error
Neutral error
DL
Left
Middle
Right
B
Target
Perseverative error
Neutral error
RL1
Right
Left
Middle
C
RL2
Left
Right
Middle
D
RL3
Middle
Left
Right
E
RL4
Right
Middle
Left
F
RL5
Left
Right
Middle
Fraction of correct entries
Block of 50 entries
WT
n
32 28 24 20 16 12 8 4
Mdx5cv
n
10 8 6 4
Mdx52
n
14 12 10 8 6 4
DMD-null
n
14 12 10 8 6 4

Fig. S7. Perseverative and neutral error curves during serial reversal tasks. *Mdx*^{5cv} (n = 9-10), *mdx52* (n = 14), *DMD-null* (n = 10-15) and WT mice (n = 32-33). Statistical tests were only performed for strains that showed deviations in learning curve of the target hole. A) Fraction of correct entries per hole. *Mdx*^{5cv} and *mdx52* mice showed increased performance during initial discrimination learning compared to WT mice ($P < 0.001$ and $P = 0.011$ respectively) and *DMD-null* mice ($P < 0.001$ and $P = 0.018$ respectively). *Mdx*^{5cv} mice showed less preference for both the middle and right holes compared to WT ($P = 0.002$ and $P < 0.001$ respectively) and *DMD-null* mice (both $P < 0.001$). *Mdx52* mice showed less preference for the right hole compared to *DMD-null* mice ($P = 0.007$). B) *Mdx*^{5cv} mice had a delayed learning curve for the initial reversal learning of the target. No differences were found in perseverative or neutral errors. C) *Mdx52* mice started the second reversal day with a higher success rate compared to WT and *mdx*^{5cv} mice ($P < 0.001$ and $P = 0013$ respectively). Additionally, *mdx52* mice showed a decrease in the perseverative error curve compared to WT and *mdx*^{5cv} mice ($P < 0.001$ and $P = 0.009$ respectively) and a decrease in neutral errors compared to WTs ($P = 0.021$). D) *Mdx52* mice showed increased performance compared to WT mice ($P = 0.018$). *Mdx52* mice showed decreased perseverative errors compared to WT mice ($P = 0.003$), while *mdx*^{5cv} mice showed decreased neutral errors compared to WT mice ($P = 0.016$). E) No significant differences were found between strains on reversal day 4. F) No significant differences were found between strains on reversal day 5.

Disease Models & Mechanisms • Supplementary information

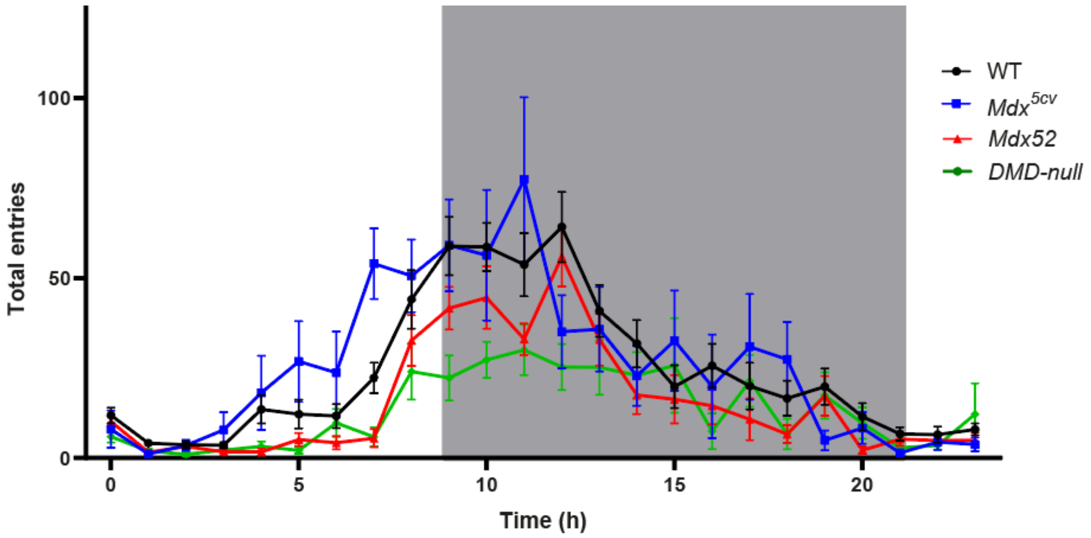

Fig. S8. Amount of entries per hour during RL1. *Mdx^5cv^* (n = 10), *mdx52* (n = 14), *DMD-null* (n = 10) and WT mice (n = 32). No statistics were performed. None of the groups showed any abrupt changes in activity over the 24h time period. Shaded area represents the dark phase.

Disease Models & Mechanisms • Supplementary information

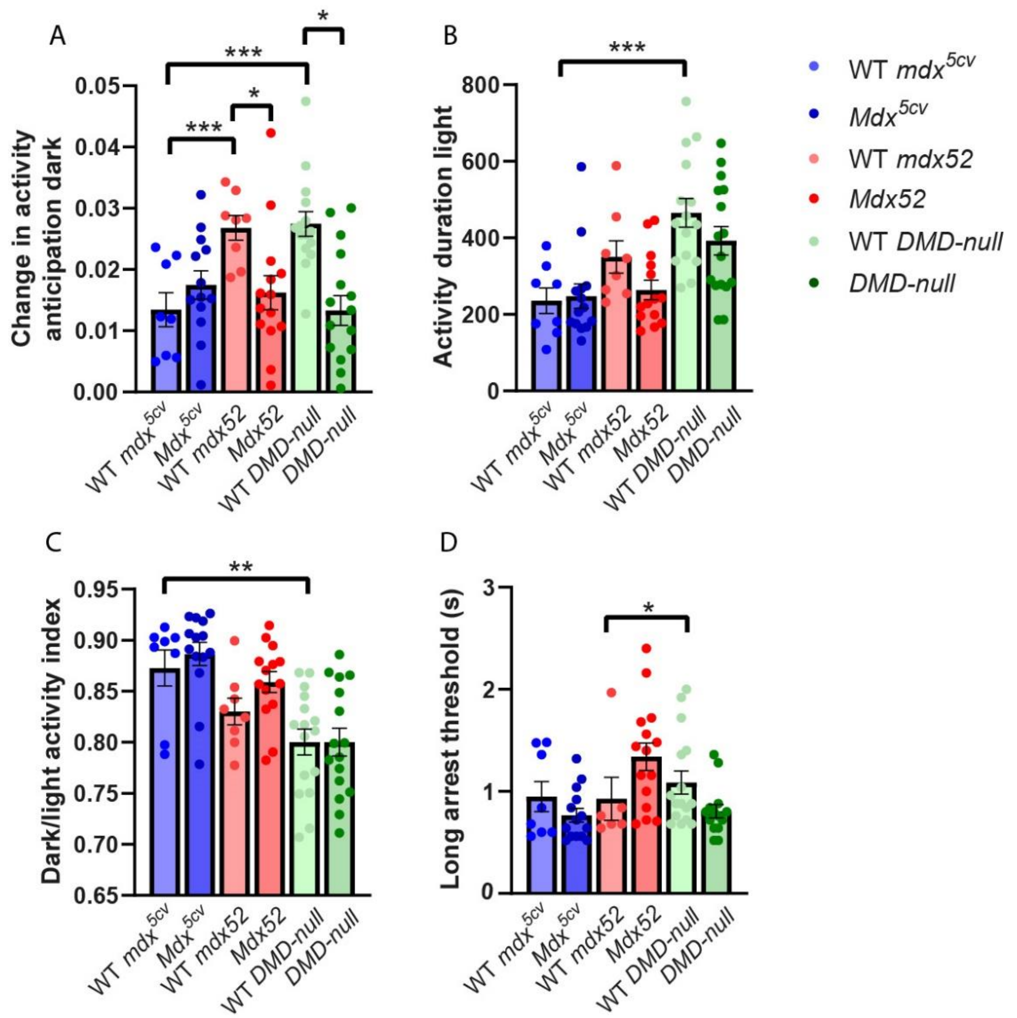

Fig. S9. Spontaneous behavior parameters in which the WT groups differed from each other. *Mdx^{5cv}* (n = 14), *mdx52* (n = 14), *DMD-null* (n = 16), *mdx^{5cv}* WT (n = 8), *mdx52* WT (n=9) and *DMD-null* WT mice (n=16) . A) *DMD-null* WTs and *mdx52* WTs showed less change in activity in anticipation of the dark phase compared to *mdx^{5cv}* WTs (both $P < 0.001$). *Mdx52* and *DMD-null* mice both showed less change in activity in anticipation of the dark phase compared to their corresponding WT groups ($P = 0.040$ & $P = 0.020$ respectively). B) *DMD-null* WTs were less active compared to *mdx^{5cv}* WTs ($P < 0.001$). C) The dark/light index was significantly lower in *DMD-null* WTs compared to *mdx^{5cv}* WTs ($P = 0.004$). D) The long arrest threshold was increased in *DMD-null* WTs compared to *mdx52* WTs ($P = 0.021$). *: $P < 0.05$, **: $P < 0.01$, ***: $P < 0.001$.

Disease Models & Mechanisms • Supplementary information

Table S1. Genotyping information. Primer sequences and primer specific PCR information used for genotyping of the mouse models.

Number	Strain	Primer sequences (3' to 5'): Forward primer Reverse primer	Annealing temperature	Number of PCR cycles	Product size
1	*Mdx*^{5cv}	TGGAGACGGAAGTAAATCTGG<br>CCTCATGAGCATGAAACTGTTC	58°C	35	147 bp
2	*Mdx*^{5cv}	Primer pair as describe above + digestion mix	58°C	35	93 bp & 54 bp
3	*Mdx52* (WT)	AGGCAACACTGCAAGATTTGGAAC<br>AACTCAAATAGATGATTGGTAAGAGGC	60°C	30	383 bp
4	*Mdx52* (Mutant)	AGGATCTCCTGTCATCTCACCTTGCTCCTG<br>AAGAACTCGTCAAGAAGGCGATAGAAGGCG	60°C	30	493 bp
5	*DMD-null* (WT)	TGGGCAAGAGTGAATTTTCC<br>ACCACCCACTTCAGGTTGAG	65°C	32	437 bp
6	*DMD-null* (Mutant)	GAATTCAGCGAGAGCCTGAC<br>GATGTTGGCGACCTCGTATT	65°C	32	453 bp

Table S2. Overview of statistical tests performed and *P*-values of WT comparisons. Differences between WT groups were found for bodyweight (WT *mdx*^{5cv} vs WT *DMD-null*: *P* = 0.008, WT *mdx52* vs WT *DMD-null*: *P* = 0.001) and during spontaneous behavior for change in activity in anticipation of the dark phase (WT *mdx*^{5cv} vs WT *mdx52*: *P* = 0.003, WT *mdx*^{5cv} vs WT *DMD-null*: *P* = 0.001). Significant differences are indicated in bold. DI: discrimination index, DL: discrimination learning, RL: reversal learning, MWM: Morris water maze, NOR: novel object placement, OP: object placement.

Test	Parameter	Statistical test	*P* value
Dark light box	Visits in light zone	One-way ANOVA	0.992
	Time in light zone	Kruskal-Wallis test	0.446
	Average time per visit	One-way ANOVA	0.174
Open field	Mean distance to wall	One-way ANOVA	0.110
	Time in inner zone	One-way ANOVA	0.339
	Distance travelled in inner zone	One-way ANOVA	0.338
	Velocity	One-way ANOVA	0.122
	Time moving	Kruskal-Wallis test	0.068
Unconditioned fear	Velocity	Kruskal-Wallis test	0.200
	Time frozen	Kruskal-Wallis test	0.208
3 chamber	DI – Trial 2	One-way ANOVA	0.575
	DI – Trial 3	One-way ANOVA	0.381
Barnes maze	Distance walked – Learning	Linear mixed models	0.674
	Distance walked – Probe	Kruskal-Wallis test	0.092
	Relative distance walked in target quadrant – Probe	One-way ANOVA	0.096
	Distance walked – Reversal learning	Linear mixed models	0.329
	Distance walked – Reversal probe	One-way ANOVA (log10)	0.618

	Relative distance walked in target quadrant – Reversal probe	Kruskal-Wallis test	0.763
	Distance walked old target – Reversal learning	Linear mixed models	0.948
	Distance walked old target– Reversal probe	Kruskal-Wallis test	0.675
	Relative distance walked in old target quadrant – Reversal probe	One-way ANOVA	0.299
	Velocity	Linear mixed models	0.534
	Interaction time target hole – Probe	One-way ANOVA	0.254
	Interaction time target hole – reversal probe	One-way ANOVA	0.639
	Interaction time old target hole – reversal probe	One-way ANOVA	0.844
MWM	Velocity	Linear mixed models	0.578
	Distance till platform – acquisition	Linear mixed models	0.661
	Distance till platform – probe	Kruskal-Wallis test	0.395
	Relative distance in target quadrant	One-way ANOVA	0.439
	Time in NE quadrant (target)	One-way ANOVA	0.794
	Time in NW quadrant	One-way ANOVA (log10)	0.508
	Time in SE quadrant	One-way ANOVA (log10)	0.723
	Time in SW quadrant	One-way ANOVA (log10)	0.296
NOR	DI – 10 min	One-way ANOVA	0.646
	DI – 24h	One-way ANOVA	0.920
OP	DI – 10 min	One-way ANOVA	0.714
	DI – 24h	One-way ANOVA	0.453
T-maze	DI – 6h	One-way ANOVA	0.351
	Alternation - 6h	One-way ANOVA	0.093
	DI – 24h	One-way ANOVA	0.875
	Alternation – 24h	One-way ANOVA	0.630
Serial reversal	DL	Linear mixed models	0.890
	RL1	Linear mixed models	0.900
	RL2	Linear mixed models	0.084
	RL3	Linear mixed models	0.290
	RL4	Linear mixed models	0.460
	RL5	Linear mixed models	0.110
Spontaneous behavior	Mean activity duration dark	One-way ANOVA	0.933
	Change in activity anticipation dark	One-way ANOVA	**0.001**
	Change in activity response dark	One-way ANOVA	0.791
	Change in activity anticipation light	One-way ANOVA	0.090
	Change in activity response light	One-way ANOVA	0.548
	Habituation index dark	One-way ANOVA	0.388
	Long shelter visit threshold	One-way ANOVA	0.506
	Cumulative duration long shelter visits	One-way ANOVA	0.772
	Long movement threshold	One-way ANOVA	0.989
	Mean long arrest duration	One-way ANOVA	0.834
Bodyweight	8-15 wks	Linear mixed models	**0.001**

Table S3. Overview of statistical parameters. Additional statistical parameters reported in case of significant differences ($P < 0.05$). Note, *P*-values reported in this table for spontaneous behavior are before false discovery rate correction. DI: discrimination index, DL: discrimination learning, MWM: Morris water maze, NOR: novel object recognition, OP: object placement, RL: reversal learning.

Test	Parameter	Statistical test	Comparison	*P*-value	Additional statistical values
Dark light box	Visits in light zone	One-way ANOVA	Overall strain comparison	0.001	F(3,75)=16.45
	Time in light zone	Mann-Whitney repeated tests	*Mdx*^{5cv} vs WT	0.001	Z=-3.54
			Mdx52 vs WT	0.001	Z=-3.53
			DMD-null vs WT	0.001	Z=-5.40
			Mdx^{5cv} vs *DMD-null*	0.043	Z=-2.03
			Mdx52 vs *DMD-null*	0.008	Z=-2.61
	Average time per visit	One-way ANOVA	Strain comparison	0.014	F(3,75)=3.80
Open field	Mean distance to wall	One-way ANOVA	Overall strain comparison	0.001	F(3,76)=11.28
	Time in inner zone	One-way ANOVA	Overall strain comparison	0.001	F(3,76)=8.92
	Distance travelled in inner zone	One-way ANOVA	Overall strain comparison	0.001	F(3,76)=11.91
	Velocity	One-way ANOVA	Overall strain comparison	0.001	F(3,76)=24.44
	Time moving	Mann-Whitney repeated tests	*Mdx52* vs WT	0.017	Z=-2.39
			DMD-null vs WT	0.001	Z=-4.88
			Mdx^{5cv} vs *DMD-null*	0.001	Z=-3.51
			Mdx52 vs *DMD-null*	0.001	Z=-2.91
Unconditioned fear	Velocity	Mann-Whitney repeated tests	*Mdx*^{5cv} vs WT	0.001	Z=-5.59
			Mdx52 vs WT	0.001	Z=-5.46
			DMD-null vs WT	0.001	Z=-5.51
			Mdx^{5cv} vs *DMD-null*	0.012	Z=-2.49
	Time frozen	Mann-Whitney repeated tests	*Mdx*^{5cv} vs WT	0.001	Z=-5.54
			Mdx52 vs WT	0.001	Z=-5.46
			DMD-null vs WT	0.001	Z=-5.51
			Mdx^{5cv} vs *DMD-null*	0.019	Z=-2.33
3 chamber	DI vs chance level – Trial 2	One sample t-test	*Mdx52*	0.024	t=-2.56
	DI vs chance level– Trial 3	One sample t-test	*DMD-null*	0.008	t=3.06
Barnes maze			WT	0.001	t=12.56

	Relative distance walked in target quadrant - Probe	One sample t-test	*Mdx^{5cv}*	0.001	t=11.05
			Mdx52	0.001	t=8.35
			DMD-null	0.001	t=7.32
	Distance walked till target – Reversal learning	Linear mixed models	*DMD-null* vs WT	0.015	F(1,215)=6.00
	Relative distance walked in target quadrant – Reversal probe	One way ANOVA	Overall strain comparison	0.029	F(3,76)=3.16
		One-sample t-test	WT	0.001	t=6.96
			Mdx^{5cv}	0.001	t=7.36
			Mdx52	0.001	t=5.64
			DMD-null	0.001	t=6.67
	Relative distance walked in old target quadrant – Reversal probe	One-sample t-test	*WT*	0.008	t=-2.837
	Velocity	Linear mixed models	*Mdx52* vs WT	0.045	F(1,150)=4.09
			DMD-null vs WT	0.001	F(1,150)=23.74
			Mdx^{5cv} vs *DMD-null*	0.010	F(1,150)=6.78
			Mdx52 vs *DMD-null*	0.019	F(1,150)=5.65
MWM	Velocity	Linear mixed models	*Mdx^{5cv}* vs WT	0.001	F(1,72)=11.74
			Mdx52 vs WT	0.001	F(1,72)=71.99
			DMD-null vs WT	0.001	F(1,72)=58.30
			Mdx^{5cv} vs *DMD-null*	0.001	F(1,72)=15.21
			Mdx52 vs *DMD-null*	0.017	F(1,72)=5.877
	Relative distance in target quadrant	One-sample t-test	WT	0.001	Z=-4.60
			Mdx^{5cv}	0.002	Z=-2.63
	Time in NE quadrant (Target)	One-way ANOVA	Overall strain comparison	0.001	F(3,73)=6.922
NOR	DI vs chance level– 10 min	One-sample t-test	WT	0.001	t=5.31
			Mdx^{5cv}	0.036	t=2.00
			Mdx52	0.004	t= 3.58
			DMD-null	0.001	t=3.76
	DI vs chance level– 24h	One-sample t-test	WT	0.001	t=4.45
			Mdx^{5cv}	0.044	t=1.39
			Mdx52	0.0015	t=2.13
			DMD-null	0.011	t=2.44
OP	DI – 10 min	One-sample	WT	0.001	t=4.82
			Mdx^{5cv}	0.001	t=4.76

Disease Models & Mechanisms • Supplementary information

		t-test	*Mdx52*	0.003	t=3.30
			DMD-null	0.001	t=5.62
	DI – 24h	One-sample t-test	WT	0.014	t=2.32
T-maze	Alternation vs chance level – 6h	One-sample t-test	*Mdx52*	0.020	t=2.81
			DMD-null	0.046	t=2.24
Serial reversal	DL	Linear mixed models	*Mdx^{5cv}* vs WT	0.001	F(1,76)=16.23
			Mdx52 vs WT	0.011	F(1,76)=6.72
			Mdx^{5cv} vs *DMD-null*	0.001	F(1,76)=31.68
			Mdx52 vs *DMD-null*	0.018	F(1,76)=5.76
	RL1	Linear mixed models	*Mdx^{5cv}* vs WT	0.017	F(1,85)=5.87
	RL2	Linear mixed models	*Mdx52* vs WT	0.001	F(1,76)=22.13
			Mdx^{5cv} vs *Mdx52*	0.013	F(1,76)=6.39
	RL3	Linear mixed models	*Mdx52* vs WT	0.018	F(1,86)=5.84
Spontaneous behavior	Mean activity duration dark	One-way ANOVA	Overall strain comparison	0.001	F(3,71)=7.22
	Change in activity anticipation dark	One-way ANOVA	Overall strain comparison	0.004	F(3,71)=4.941
	Change in activity response dark	One-way ANOVA	Overall strain comparison	0.016	F(3,71)=3.65
	Change in activity anticipation light	One-way ANOVA	Overall strain comparison	0.032	F(3,71)=3.09
	Change in activity response light	One-way ANOVA	Overall strain comparison	0.001	F(3,71)=8.70
	Habituation index dark	One-way ANOVA	Overall strain comparison	0.001	F(3,71)=6.98
	Long shelter visit threshold	One-way ANOVA	Overall strain comparison	0.001	F(3,71)=6.50
	Cumulative duration long shelter visits	One-way ANOVA	Overall strain comparison	0.001	F(3,71)=6.51
	Long movement threshold	One-way ANOVA	Overall strain comparison	0.001	F(3,71)=10.32
			DMD-null vs WT	0.014	Z=-2.43

	Mean long arrest duration	Mann-Whitney repeated tests	*Mdx*^{5cv} vs *DMD-null*	0.009	Z=-2.60
Bodyweight	8-15 wks	Linear mixed models	*Mdx52* vs WT *mdx52*	0.017	F(1,22)=6.64
			DMD-null vs WT *DMD-null*	0.001	F(1,29)=36.88

Disease Models & Mechanisms • Supplementary information
